# 3-*O*-sulfated heparan sulfate interactors target synaptic adhesion molecules from neonatal mouse brain and inhibit neural activity and synaptogenesis in vitro

**DOI:** 10.1038/s41598-020-76030-4

**Published:** 2020-11-05

**Authors:** Auriane Maïza, Nazha Sidahmed-Adrar, Patrick P. Michel, Gilles Carpentier, Damien Habert, Carine Dalle, Walid Redouane, Magda Hamza, TH van Kuppevelt, Mohand Ouidir Ouidja, José Courty, Sandrine Chantepie, Dulce Papy-Garcia, Olivier Stettler

**Affiliations:** 1 Gly-CRRET-Croissance Cellulaire, Réparation et Régénération Tissulaire, Univ Paris Est Creteil, Gly-CRRET, F-94010 Creteil, France; 2 Paris Brain Institute, CNRS UMR 7225, INSERM U 1127, Sorbonne Université, Paris, France; 3 Department of Biochemistry, Radboud University Medical Centre, Nijmegen, The Netherlands

**Keywords:** Neuroscience, Development of the nervous system, Synaptic development

## Abstract

Heparan sulfate (HS) chains, covalently linked to heparan sulfate proteoglycans (HSPG), promote synaptic development and functions by connecting various synaptic adhesion proteins (AP). HS binding to AP could vary according to modifications of HS chains by different sulfotransferases. 3-*O*-sulfotransferases (Hs3sts) produce rare 3-*O*-sulfated HSs (3S-HSs), of poorly known functions in the nervous system. Here, we showed that a peptide known to block herpes simplex virus by interfering with 3S-HSs in vitro and in vivo (i.e. G2 peptide), specifically inhibited neural activity, reduced evoked glutamate release, and impaired synaptic assembly in hippocampal cell cultures. A role for 3S-HSs in promoting synaptic assembly and neural activity is consistent with the synaptic interactome of G2 peptide, and with the detection of Hs3sts and their products in synapses of cultured neurons and in synaptosomes prepared from developing brains. Our study suggests that 3S-HSs acting as receptors for herpesviruses might be important regulators of neuronal and synaptic development in vertebrates.

## Introduction

Heparan sulfate proteoglycans (HSPG) act as central synaptic organizers by interacting to extracellular matrix (ECM)-located and plasma membrane-associated molecules^1–11^. This function implicates the heparan sulfate (HS) chains covalently associated to HSPG protein core, since HSs removal disrupts synaptic assembly^6,11^. HSs may regulate the signaling of HSPG by orchestrating their interactions with a wide variety of membrane-bound and soluble heparin-binding proteins (HBP)^12^. Different studies on various cellular systems have provided evidences that functional specificities of HSs in mediating ligand interactions may arise from unique modification of their disaccharide backbone, in particular by *N*- and *O*-sulfations^12,13^. However, the consequences of these modifications on neuronal and synaptic functions are still poorly evaluated, especially in vertebrates. Among HSs modifications, a rare 3-*O*-sulfation producing specific 3-*O*-sulfated HSs (3S-HSs) of unknown functions are assured by HS 3-*O*-sulfotransferases (Hs3sts)^13^. Individual Hs3st can produce, within HSs having specific prior modifications (i.e. by combinations of *N*-, 2-*O*-, and 6-*O*- sulfations), unique protein-binding sites for Antithrombin (At)^14^, herpes simplex virus-1 (HSV-1) glycoprotein D (gD)^15^, Cyclophilin-B^16^ and for Neuropilin-1 (Np1)^17^. Accordingly, the isoform Hs3st1 creates an At-site on HSs^13^ but is unable to produce a gD one^18^. Conversely, the brain-enriched Hs3st2 and Hs3st4^19^ produce a HSV-1 binding gD-site but not an At one. While several Hs3Sts, including Hs3st2 and Hs3st4, are preferentially expressed at the time of synaptic formation in the mouse brain^19^, it is not known whether 3S-HSs are present at the synapse and/or if they impact neural functions and signaling in mice. Establishing this is fundamental because HSs and 3S-HSs have been associated to pathological processes impacting synapses, including HSV-1 infections^20^, and psychiatric diseases such as Alzheimer’s disease (AD)^21,22^ and autism spectrum disorders (ASDs)^2,11,23–25^. Analysis of the function of 3S-HS by means of *Hs3st* invalidations is made difficult however by the expected compensation between the Hs3st forms generating the same modifications of 3-*O*-sulfate. Alternatively, studies on HSV-1 lifecycle may provide specific clues and tools to get insights on the 3S-HSs interactome and signalosome in the nervous system. HSPG are required for HSV-1 cell attachment and Hs3sts and their product 3S-HSs mediate HSV-1 cell entry through the viral protein gD^15,18,20,26^. Strikingly, recent data support the hypothesis that HSV-1 may contribute to the onset of AD by inducing the accumulation of amyloid Aß peptides and of hyperphosphorylated Tau, two hallmarks of AD degeneration in the brain^27^. Since 3S-HSs also contributes to hyperphosphorylation of Tau in a model of AD-related tauopathy^21^, the HSV-1 infection through 3S-HSs may reveal important signaling components associated to pathways linked to specific HS types in physiological and pathological contexts.

To characterize the signaling pathways and function of 3S-HS moieties during neuronal development, we performed proteomic and functional experiments using inhibitory peptides of HSV-1 entry allowing to distinguish 3S-HSs from non-3-*O*-sulfated HSs. G1 and G2 peptides have been previously isolated by phage display technologies either against HSs lacking 3-*O*-sulfates (G1 peptide) or against 3S-HS (G2 peptide)^26,28^. Both peptides block HSV-1 cellular entry but G2 specifically by preventing the association of gD-type 3S-HSs and the viral gD protein which is necessary for viral fusion with host cell^26,29^. Correlatively, G2 inhibitory effect on HSV-1 entry is tributary to Hs3sts expression in cells^28^, linking G2 effect to the enzyme producing 3S-HSs. By relying on the specific binding of G2 on HSs at the cell surface^30^, and after having found here that 3S-HSs and two Hs3sts producing gD-type 3S-HSs were expressed at synapse, we established a comparison of the synaptic interactome of G1 and G2 peptides, and of their effects, at the synaptic and neuronal network levels. Our approach allowed to characterize different proteins of the gD-type 3S-HS signaling pathway(s) linked to the life cycle of HSV-1 and/or involved in synaptic organization. The importance of these pathways, never described at this level for a modification of HS, is outlined by specific inhibitory effects of anti-3S-HSs peptides on developing neuronal cells.

## Results

### 3S-HSs and their enzymes are localized at central synapse

**Figure 1:**
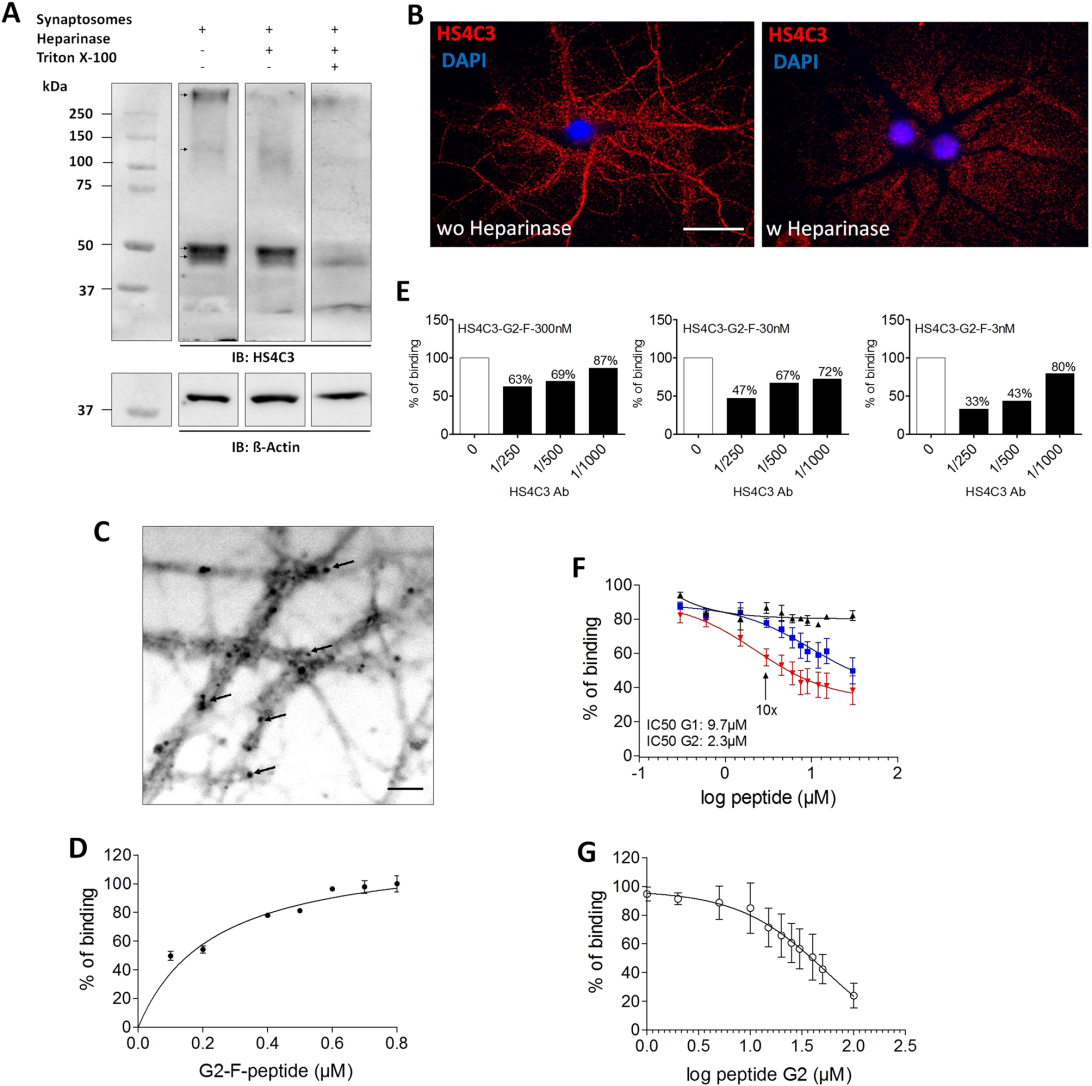
Neuronal and synaptic expression of 3S-HS. (**A**) molecular species recognized by HS4C3 Ab in PND7 synaptosomal fractions ± heparinase or Triton-X100. Shown is a representative Western Blot. Tracks from a same blot have been cropped by removing the empty interspersed tracks (original blot is shown as supplementary Fig. S2, Supplementary file). Immunoreactive species are pointed by arrows on blots. (**B**) confocal image of fluorescent hippocampal neurons (DIV20) ± heparinase and immunolabelled with HS4C3 Ab. (**C**–**G**) characteristics of G2 peptide binding. (**C**) G2 AlexaFluor (G2-F) peptide binding on hippocampal cell neurites. The fluorescent image has been grayscale to enhance the contrast. Arrows point to fluorecent dots. (**D**) G2-F binding assay to heparin (Hp) under increasing concentrations of G2-F (n = 3 independent experiments). (**E**) binding of G2-F at 3, 30 or 300 nM to Hp is inhibited by increasing concentration of HS4C3 Ab. (**F**) competition assay for the binding of G2-F to Hp with increasing concentration of unlabeled G2 (red), G1 (blue) or Cp (black) (n = 3 independent experiments); arrow pointed G2 concentration 10 × higher than that of G2-F. (**G**) competition assay for the binding of G2-F to synaptosomes by using increasing concentrations of unlabeled G2 peptide (n = 3 independent experiments). Bar in (**B**), 30 µm, bar applies for the two panels. Bar in (**C**), 5 µm.

**Figure 2:**
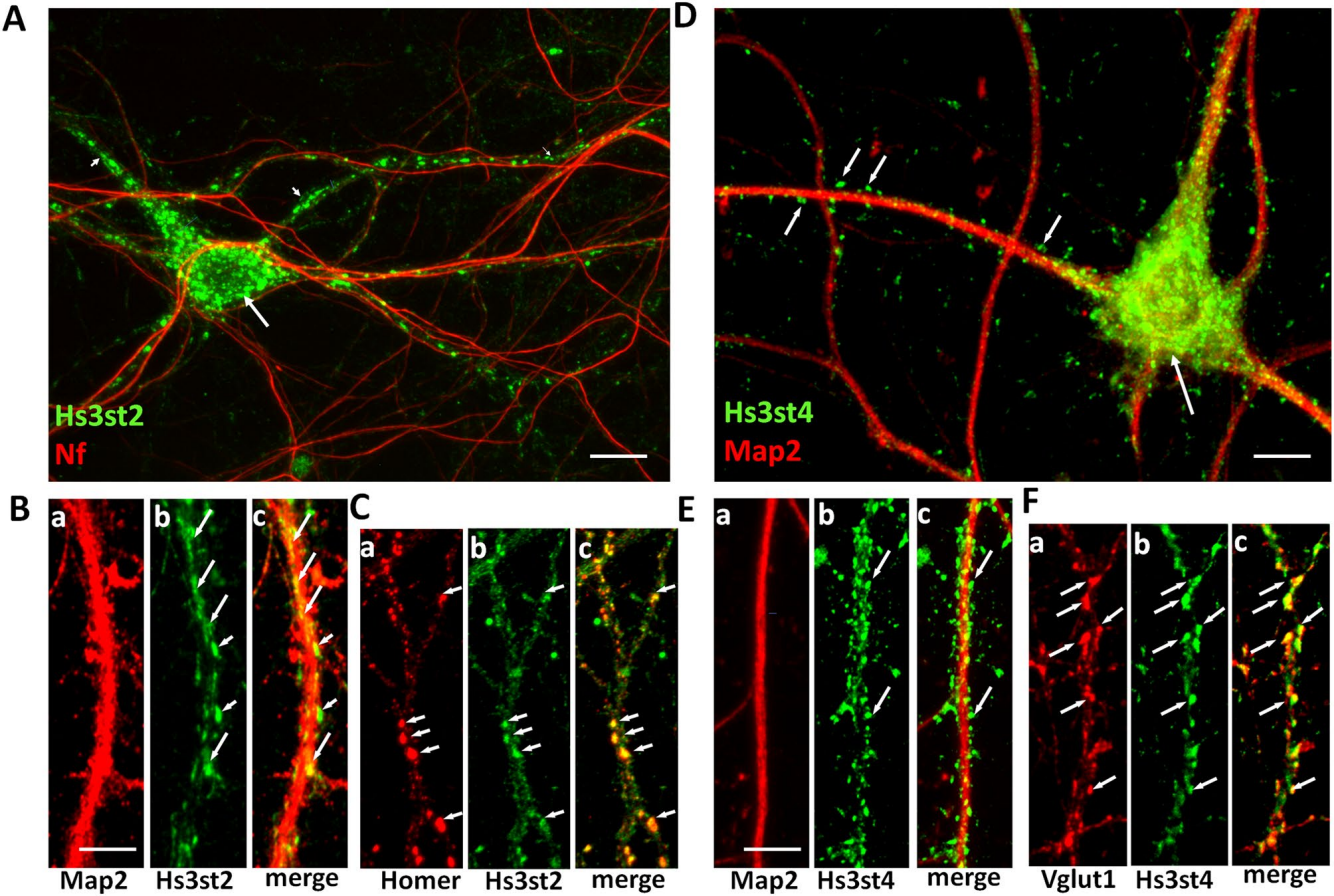
Subcellular distribution of Hs3st2 and Hs3st4 in permeabilized primary hippocampal neurons. (**A**) Hs3st2 Ab generates IF clusters (in green) within neuronal cell bodies (long arrow) and dendrites (short arrows) but not in axons as indicated by co-IF with the axonal marker neurofilament (Nf, in red). (**B**) high magnifications of neurites co-immunolabelled for Hs3st2 (in green) and the dendritic marker Map2 (Ba-c, in red). Numerous Hs3st2 IF clusters colocalize with Map2 in dendrites (long arrows, Bb,c), some are observed at the margin of Map2 immunolabelling (short arrows, Bb,c). (**C**) Hs3st2 IF clusters (in green) colocalize with the postsynaptic marker Homer (in red) along dendrites (Ca-c, arrows). (**D**) Hs3st4 Ab generates immunofluorescent puncta (in green) that seem to surround Map2-immunoreactive dendrites (in red, short arrows) and the cell bodies (long arrow). (**E**) high magnifications of neurites co-immunolabelled with Hs3st4 (in green) and Map2 (in red, Ea-c). Hs3st4 immunofluorescent puncta (in green) surround Map2 immunolabelled dendrites. (**F**) Hs3st4 puncta (in green) colocalize with Vglut-1 immunoreactive clusters (in red, arrows in Fa-c). Bars in (**A**), 20 µm, in (**B**), 5 µm (applies for** C** and** F**), in (**D**), 10 µm and in E, 8 µm.

The expression peak of Hs3st2 and Hs3st4, two enzymes producing 3S-HS, during synaptogenesis in the mouse brain^19^, suggested a role for 3S-HSs in relation to synapses. To explore it, we first analyzed the expression of 3S-HSs at synapse by using, the HS4C3 antibody that recognizes over sulfated HS and preferentially 3S-HSs^31^, Western-immunoblot (WB), and purified synaptosomal fractions enriched in presynaptic marker Vglut1, and in postsynaptic marker PSD-95 (Fig. S1, Supplementary file). HS4C3 Ab detected several immunoreactive bands in synaptosomes lining up approximately at 50 kDa, another band above 250 kDa, and a smear between 110 and 200 kDa (Fig. 1A). Overnight treatment of non-permeabilized synaptosomes with heparinase digesting HSs chains completely removed the highest molecular weight band but only reduced the other ones (Fig. 1A). However, overnight treatment with heparinase combined with permeabilization of synaptosomes removed almost all the HS4C3 immunoreactive signal (Fig. 1A). This indicated that while 3-*O*-sulfates and other sulfated groups may be present at the synaptosomal surface, some intrasynaptic sulfated chains exist, and became accessible to heparinase digestion only after synaptosomes permeabilization. In agreement with the classical extracellular location of HSs, HS4C3 Ab also recognized heparinase-sensitive sulfated epitopes at the surface of non-permeabilized hippocampal neurons in vitro, both at the cell body and at the neuritic levels (Fig. 1B). In cell culture, heparinase produced a displacement of the punctiform labeling generated by HS4C3 Ab from the cell surface to the ECM surrounding the treated neurons (Fig. 1B). We interpreted this shift as the result of sulfated HSs chains shedding from the cell surface and their attachment to cationic poly-d-lysine used for the coating on coverslips and/or to other secreted molecules in the ECM. G2-F, a fluorescent form of G2 peptide recognizing gD-type 3S-HSs^26^ and which binds to heparan sulfates onto cell membrane in vitro^30^ produces, as HS4C3 Ab, a punctiform labelling at the surface of hippocampal cell neurites (Fig. 1C). By ELISA, we observed that G2-F dose-dependently bound to over-3-*O*-sulfated heparin (Hp) (Fig. 1D), while this binding is dose-dependently prevented by HS4C3 Ab in line with the preferential recognition of 3S-HSs by HS4C3 Ab (Fig. 1E)^31^. In addition, G2-F competed with unlabeled G2 for its binding to Hp with an IC50 lower than that of G1 peptide that recognizes HS lacking 3-*O* sulfates (Fig. 1F), confirming that G2 and G1 recognized distinct sites on HS^26^. Finally, we found that G2 bound to synaptosomes in a competitive manner (Fig. 1G) in agreement with our observation that synaptic fractions contain specific 3-*O*-sulfated sites. To further evaluate the functional relevance of our detection of 3S-HSs in neurons and synaptosomes, we then examined the expression of neuronal Hs3sts (Fig. 2). In mature (DIV20) permeabilized neurons, Hs3st2 Ab produced fluorescent clusters of various sizes ranging from puncta to larger vacuolar-like structures (Fig. 2A). These fluorescent clusters filled the neuronal cell body and the dendrites but not the axons, as revealed by co-IF with the axonal marker neurofilament (Nf). At higher magnification (Fig. 2B), most Hs3st2-IF clusters seemed colocalized with Map2 indicating their proximity to the internal dendritic compartment (Fig. 2Ba–c). A number of these IF clusters matched the distribution of the dendritic spine marker Homer (Fig. 2Ca–c) whereas they co-localized only weakly with the presynaptic markers Vglut-1 or Sv2 (Supplementary Fig. S3, Supplementary file). Collectively thus, the co-IF of Hs3st2 and distinct neuronal markers indicated a preferential localization of Hs3st2 in the postsynaptic compartment. Interestingly, Hs3st4 Ab displayed a different IF pattern in the same conditions of cell fixation and permeabilization (Fig. 2D–F). Hs3st4 puncta distributed apparently around, rather than inside, dendrites as for Hs3st2 (Fig. 2D,Ea–c) and colocalized with Vglut-1 IF indicating the presence of Hs3st4 in presynaptic elements along dendrites (Fig. 2Fa–c). The present IF experiments thus provided evidences that synapses have the potential to generate 3S-HSs from the dendrite with Hs3st2 and from the axon with Hs3st4.

**Figure 3:**
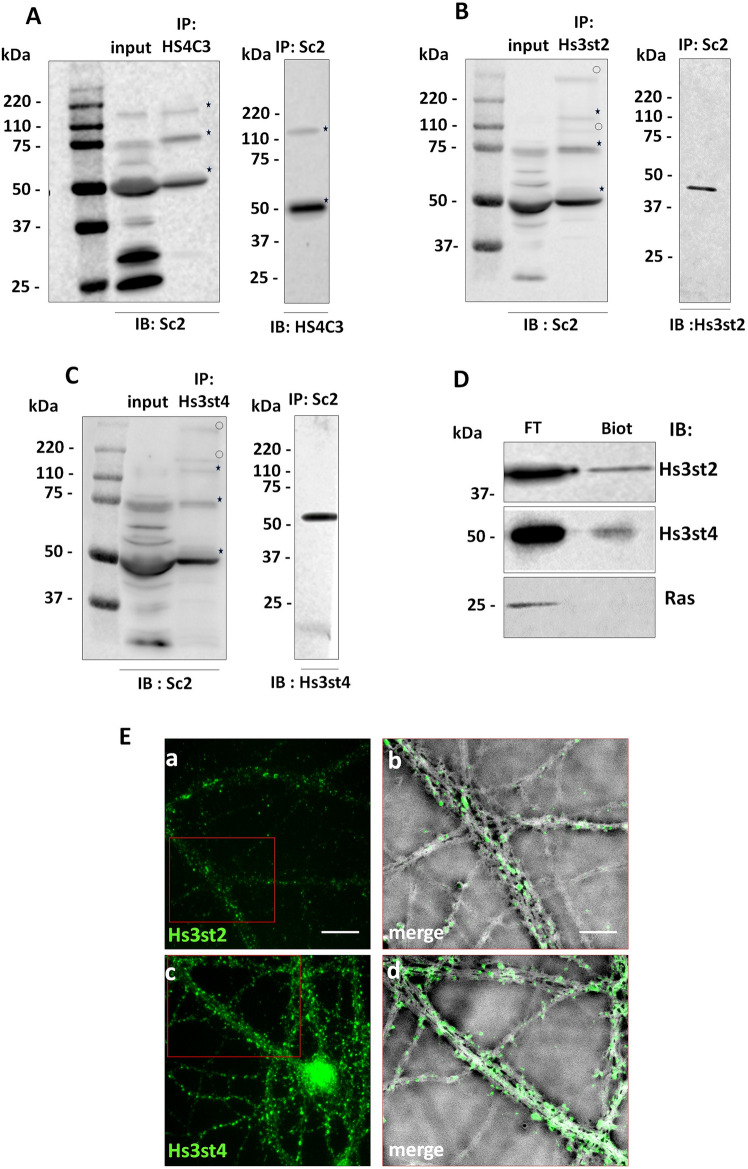
Expression of 3S-HS and Hs3sts at the synaptic surface. (**A**) co-immunoprecipitation of 3S-HS and Sdc2 from the surface of synaptosomes. HS4C3 and Sdc2 Abs co-immunoprecipitate molecular species migrating at 50 kDa and between 110 and 220 kDa. (**B**,**C**) Sdc2 is co-immunoprecipitated with Hs3st2 (**B**), and Hs3st4 (**C**) from the synaptosomal surface. (**D**) synaptosome biotinylation indicates the presence of Hs3st2 and Hs3st4 at the synaptic membrane surface. The stars in (**A**–**C**) mark the immunoprecipitated molecular species common for HS4C3, Hs3st2 and Hs3st4 Abs, the clear circles mark the molecular species that are different. The Ras immunoreactive band in (**D**) is shown as a negative control of cell surface biotinylated proteins. (**E**) Hs3st2 and Hs3st4 localization in non-permeabilized mature primary hippocampal neurons (DIV 20). Ea, c immunofluorescent labelling for Hs3st2 and Hs3st4 (in green) are observed at the surface of mature hippocampal cell neurites. Eb, d, high magnification of insert in red in (Ea) and (Ec) respectively showing immunofluorescence on bright field views. Hs3st2 and Hs3st4 immunolabelling appear punctiform (green puncta). Bar in (Ea), 8 µm (applies for Ec). Bar in (Eb), 4 µm (applies for Ed). IP, immunoprecipitation, IB, immunoblotting.

Since HSs are part of HSPG, we further examine by co-immunoprecipitation (co-IP) whether Syndecan-2 (Sdc2), a major synaptic HSPG^32^ could carry 3S-HSs. HS4C3 Ab co-immunoprecipitated from non-permeabilized synaptosomes three main molecular species from the synaptosomal surface that were recognized by an Ab directed against Sdc2: one at 50 kDa, a second at 75 kDa, and another one located between 110 and 220 kDa (Fig. 3A). Additional molecular species reacting with Sdc2 Ab, but not recognized by HS4C3 Ab or not accessible to this Ab possibly because intracellular, were observed migrating between 50 and 75 kDa, at 25 kDa, at 37 kDa, and between 25 and 37 kDa (Fig. 3A). Reverse experiments by using the Sdc2 Ab for IP, and the HS4C3 Ab for WB, confirmed that at least the 50 kDa and the band above 110 kDa were related to Sdc2 (Fig. 3A). The co-IP experiments indicated that some Sdc2 species, most likely monomers at 50 kDa, and oligomeric forms above 50 kDa which are stable in SDS-PAGE^33^, were recognized by HS4C3 Ab and thus carry multisulfated and 3-*O*-sulfated HSs. Interestingly, Sdc2 Ab also co-immunoprecipitated the sulfotransferases Hs3st2 and Hs3st4 from the synaptosomal surface (Fig. 3B,C). This Hs3sts extracellular location is consistent with a similar recent observation^34^ and was confirmed by biotinylating assay from purified synaptosomes (Fig. 3D) and by IF from non-permeabilized hippocampal cells (Fig. 3E) indicating that the Golgi enzymes Hs3sts^13^ may also have extracellular location at the synapse and that they might interact with Sdc2 through their 3S-HSs chains.

### HSV-1 inhibitory peptides G1 and G2 target synaptic HSPGs and HBP molecules

**Figure 4:**
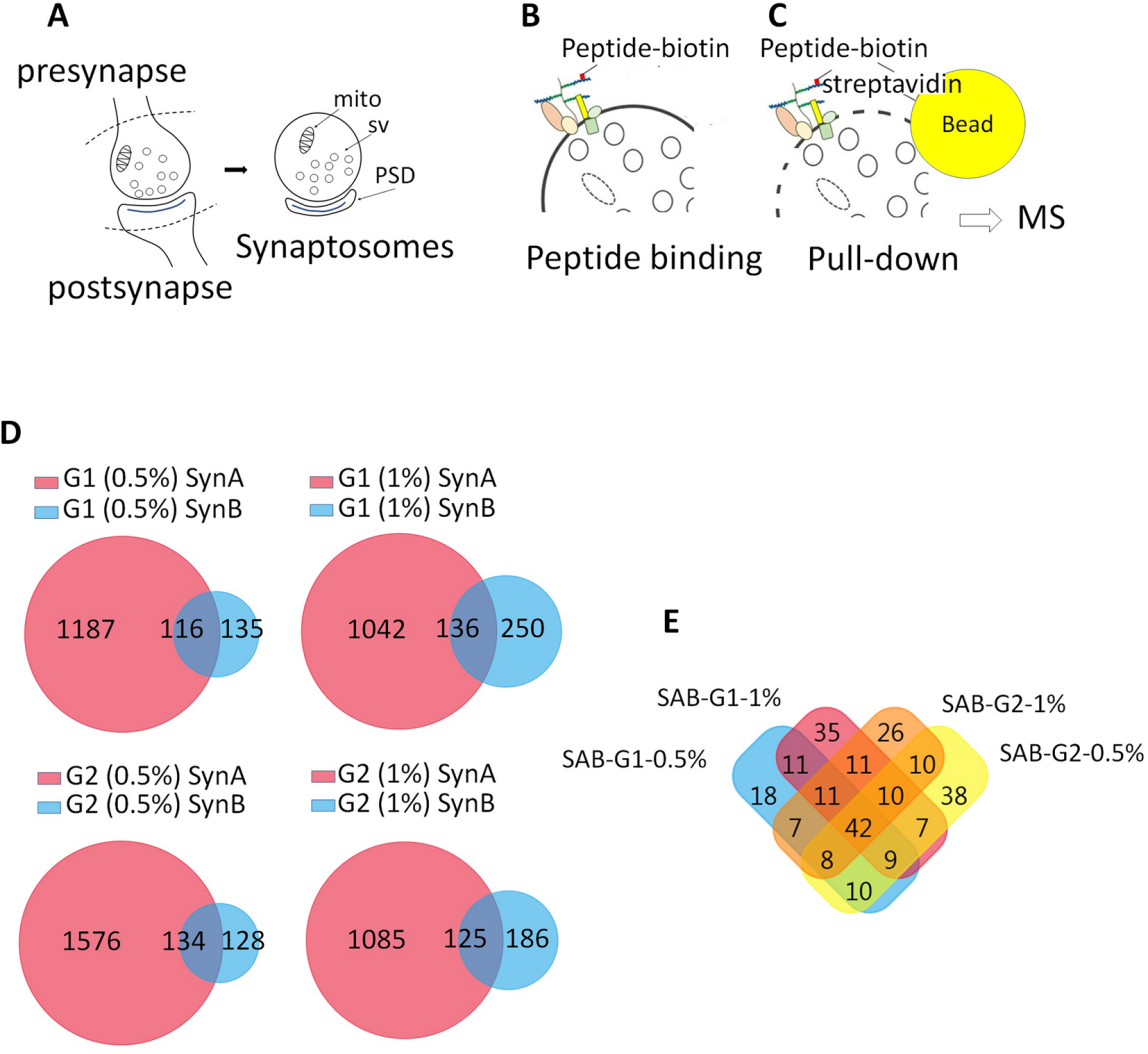
HS- and 3S-HS-related interactome identified from PND9 forebrain synaptosomes. (**A**) graphical sketches of synaptosomes prepared from central synapses. (**B**) graphical sketches of binding of biotinylated G1 and G2 peptides on synaptosomes before their lysis. (**C**) recovery and analysis by MS of HSs binding proteins after synaptosome lysis, by using streptavidin beads and their affinity for HS-linked biotinylated peptides. (**D**) Venn diagrams of proteins analyzed by MS and recovered with G1 or G2 after synaptosome lysis with either 0.5% or 1% Triton-X100. SynA and SynB stand for two independent synaptosome preparations with SynB having half the protein concentration of SynA. Shown are the numbers of proteins identified for each sample. Intersections show the proteins common to SynA and SynB (i.e. “SAB” in text and Table S2). (**E**) Venn diagrams of proteins “SAB” as defined in (**D**). mito, mitochondria, sv, synaptic vesicles, PSD, postsynaptic density.

Having documented the synaptic expression of two Hs3sts (i.e. Hs3st2 and Hs3st4) known to produce 3S-HSs receptors for HSV-1^13^ (i.e. gD-type 3S-HS), and the association of HS recognized by HS4C3 Ab with Sdc2, we continued by exploring the interactions of sulfated gD-type 3S-HS at the synapse through a randomized analysis. For this, we used a pull-down assay approach (see Supplementary file) using biotinylated peptides and allowing to precipitate membrane receptors together with their intracellular associated proteins. G2 peptide was used as a bait, rather that HS4C3 Ab, because this later molecule also recognizes an At-type 3S-HS^31^, not synthetized by Hs3st2 and Hs3st4^13^. We precipitated biotinylated G1 and G2 peptides interacting with HSs lacking 3-*O*-sulftates (peptide G1) or with gD-type 3S-HSs (peptide G2)^26^ on the surface of mouse synaptosomes (Fig. 4A,B). Pull-down of biotinylated-G1 or -G2, using streptavidin beads and two different lysis conditions (0.5 and 1% Triton-X100) to optimize protein recovery, allowed to recover peptide-bound HSPG linked to extra- and intracellularly associated proteins (Fig. 4B,C). By this way, we recovered hundreds of proteins, which were identified by mass spectrometry (Fig. 4C) (data depository file “G1G2 synaptosome interactome”, Supplementary file). Synaptosomal fractions not incubated with peptides served as control and proteins that non-specifically bound to streptavidin beads were removed from the analysis to generate a normalized protein collection (data depository file “G1G2 synaptosome interactome-normalized”, Supplementary file). Using the proteomic software FunRich, we compared normalized protein samples from two independent pull-down experiments, SynA and SynB, which differed on the input protein concentration (input in SynA was twice concentrated compared to SynB) (Fig. 4D). As expected, we found that halving the protein concentrations in the input decreased the total amount of protein recovered in SynB compared to SynA (Fig. 4D). However, all the samples processed in SynB (G1 or G2 combined with either 0.5 or 1% Tx-100) appeared also enriched in proteins common to the SynA and SynB experiments (i.e. “SAB” at the intersections of Venn diagrams in Fig. 4D). We used theses “SAB” proteins appearing as the most specific for subsequent proteomic analysis (data depository file “SAB”, Supplementary file). The proteins in “SAB” recovered both by G1 and G2 were the more numerous (approximately 45% of total proteins), followed by proteins recovered by G2 only (approximately 29% of total proteins), and proteins recovered by G1 only (approximately 25% of total proteins) (Fig. 4E). Membrane proteins in “SAB” collections formed a subgroup of thirty proteins (about 12% of total) containing three major HSPG, namely Neurexin-1a and -1b (Nrxn-1a, Nrxn-1b) and Glypican-1 (Gpc-1), four Nrxn-binding proteins, that is, Neuroligins 2, 3 and 4 (Nlgns 2–4), Latrophylin-1 (Agrl1), and five heparan/heparin binding proteins, namely Nlgns 2–4, Thy-1, and protein tyrosine phosphatase receptor type D (Ptprd) (Table 1). We established a ranking based on the MSMS identification of these membrane proteins in either 4, 3, 2 or 1 out of 4 samples (SynA-G1/G2-0.5% Triton X-100; SynA-G1/G2-1% Triton X-100; SynB-G1/G2 0.5% Triton X-100; SynB-G1/G2 1% Triton X-100). Many of these membrane proteins, including the HSPG Nrxn-1a, the Nrxn ligands Nlgns, and Agrl1, were identified in samples pulled-down both with G1 and G2 peptides. However, when synaptosome concentration was reduced (i.e. in SynB versus SynA), some membrane proteins were not detected by MSMS in protein samples recovered either by G1 or G2 (Table 1) suggesting a difference in efficiency of peptides in their ability to recover specific target proteins. At1b2, mGlur2, and Ptprg appeared for instance more readily recovered by G1, Nrxs, Nlgns, Gpc1 by G2, while some proteins were still identified both by G1 and G2 (e.g. Agrl1; Ptprd; N-cadherin, i.e. Cadh2) (Table 1).

**Table 1:** PLGS peptide score (Waters) of membrane proteins recovered by G1 or G2 peptides according to protein concentration in the input [SynA (SA) = 2× SynB (SB)] and to Triton X-100 concentrations (0.5% or 1%).

Input concentration	2× protein		2× protein		1× protein		1× protein	
Protein name	SA G1-0.5%	SA G1-1%	SA G2-0.5%	SA G2-1%	SB G1-0.5%	SB G1-1%	SB G2-0.5%	SB G2-1%
Nrxn1a	2374	3441	3552	2171	–	136	306	1150
Gpc1	389	–	1287	230	–	–	1367	1050
Nrxn1b	1416	2101	1917	1173	–	–	–	779
Nlgn3	1150	1002	1615	346	1022	–	1063	640
Nlgn2	920	854	1918	427	998	–	802	635
Cadh2	396	437	647	258	923	651	1825	598
L1cam	233	–	–	176	264	–	623	587
Nlgn4	732	667	1281	–	872	–	756	537
Agrl1	906	931	924	603	588	316	1147	530
Slc12a5	1443	1278	1446	451	–	–	288	377
Gria2	1476	1624	2439	536	700	331	–	343
Ptprd	775	762	676	212	589	280	244	312
Plxna4	1666	1367	1711	1214	–	–	–	260
Plxnb2	621	611	446	231	–	–	–	253
Plxna1	1701	1622	2142	1125	–	–	–	237
Fam171a2	389	–	–	269	–	176	261	236
Gabarb1	499	661	1651	404	391	–	302	215
Thy1	1478	–	566	559	–	1288	1961	204
Plxna2	1246	1319	1302	1008	–	–	–	202
Nptn	944	326	943	633	–	–	–	–
Epha4	1086	1837	1939	379	252	–	–	–
Tmeff1	398	263	1395	–	–	–	341	–
Lsamp	501	–	–	245	–	–	–	–
At1b2	7069	3063	5960	2124	–	265	–	–
Ca2d1	1689	608	976	245	287	–	702	–
mGlur2	305	250	–	–	–	189	–	–
Ptprg	–	223	–	–	–	403	–	–
Glur1	720	733	693	223	306	–	324	–
mGlur3	689	710	527	308	–	219	240	–
Cad13	655	435	512	280	381	–	293	–

### Targeting 3S-HSs identifies specific synaptic biological processes

Through the pull-down approach, our expectation was to recover, not only HSPG and associated membrane proteins, but also intracellular molecules associated to their signaling pathways (as sketched in Fig. 4B,C)^6,35–37^. Accordingly, we found that more than four fifth of the proteins collected with biotinylated G1 and G2 were intracellular, some of them belonging to the Nrxn pathway (Cask, Elks, Band 4.1)^38–41^. Examples of intracellular molecules sorted by G1 and/or G2 included kinases (Casein kinases, CdK5), phosphatases (Ppp3r1), rho GTPase-related proteins (Cdc42, Arhgap1), molecular motors (dyneins and kinesins), cytoskeletal associated proteins (myosins, Tau, Arp, septins), proteins involved in exocytosis/endocytosis processes (exocyst components, Snap25, Clta, Ap2, Picalm-1), as well as proteins involved in neurodegenerative pathologies such as AD (Tau, Picalm-1). Because G1 and G2 interfere with HSV-1, we also wondered what the representation of proteins functionally linked to HSV-1 was in our screening. This represented 17% of total proteins in “SAB” (Supplementary Table S1, Supplementary file) and included distinct categories of proteins participating in the lifecycle of HSV-1. On a functional basis, these proteins related to the actomyosin system such as Myosins and Drebrin, to proteins kinases such as Cdk5, to proteins involved in microtubule based transport and dynamics such as Dynein, Tau and Clasp1, to proteins implicated in vesicular trafficking such as Snap25 and Picalm-1, to the Wnt signaling pathway (i.e. catenins), and strikingly to cellular proteins involved in HSV-1 replication including Dnaj, Tera/Vcp, Banf1, and Importin (Imp). The diversity of signaling molecules identified by G1 and/or G2 was in line with the plurality of signaling pathways associated to HSPG at the synapse^6,11,35^. Nevertheless, we tried to determine which endogenous signaling processes were better represented in PND9 synaptosomes from the protein reproductively recovered with G2, in comparison to these recovered with G1. For this, we used the enrichment tool of FunRich to analyze first the enrichment of the G1 or G2 “SAB” fractions in cellular components. Then, we repeated the analysis for the enrichment in biological processes (Supplementary Tables S2A, B, Supplementary file). G1 and G2 “SAB” protein fractions appeared enriched in the same types of synaptic components when compared to the Uniprot rodent protein database (Supplementary Table S2A, Supplementary file). Gene ontology (GO) terms that occurred preferentially and significantly were for instance “presynaptic membrane”, “glutamatergic synapses”, “gabaergic synapses”, “postsynaptic density”, “dendritic spine” and “synaptic vesicle”. This suggests that G1 and G2 had the same ability to concentrate synaptic proteins from synaptosomes. However, G1 and G2 fractions appeared comparatively different after analyzing their enrichment in proteins involved in specific biological processes (Supplementary Table S2B, Supplementary file). G2, but not G1, displayed an enrichment in proteins implicated in “cell adhesion”, “postsynaptic membrane assembly”, “synaptic vesicle clustering”, and “synaptic vesicle endocytosis”. By comparison, G1, but not G2, displayed an enrichment in proteins involved in “modulation of chemical synaptic transmission”, “microtubule-based movements” and “anterograde dense core vesicle transport”. Finally, both G1 and G2 produced protein fractions enriched in “synapse organization” and “presynaptic membrane assembly”. Because G2 relation to 3S-HS and G1 to HS lacking 3-*O*-sulfates^26^, this suggested that 3S-HS and HS may participate to distinct biological events though overlapping synaptic processes. We next compared the biological processes of “SAB” proteins recovered by G2 with the predicted biological processes involving Hs3st2 and Hs3st4 as analyzed by GeneNetwork v2.0 (https://genenetwork.nl/)^42^. GeneNetwork, which uses gene co-regulation to predict pathway membership by integrating 31,499 public RNA-seq sample, provided predictions consistent to ours (Supplementary Tables S3A, B, Supplementary file), since “transmission across synapses” appeared as a major function deduced from the Hs3st2 and Hs3st4 reactomes. Given in addition that loss of 3-*O*-sulfation in invertebrates generate abnormal synaptic vesicles distribution^9^, and our finding of an association between G2 and the ontology term “synaptic vesicle clustering”, we tested the effect of G2 on neural activity and exocytosis.

### Acute inhibition of 3S-HSs by G2 disrupts neuronal activity and reduces exocytosis

**Figure 5:**
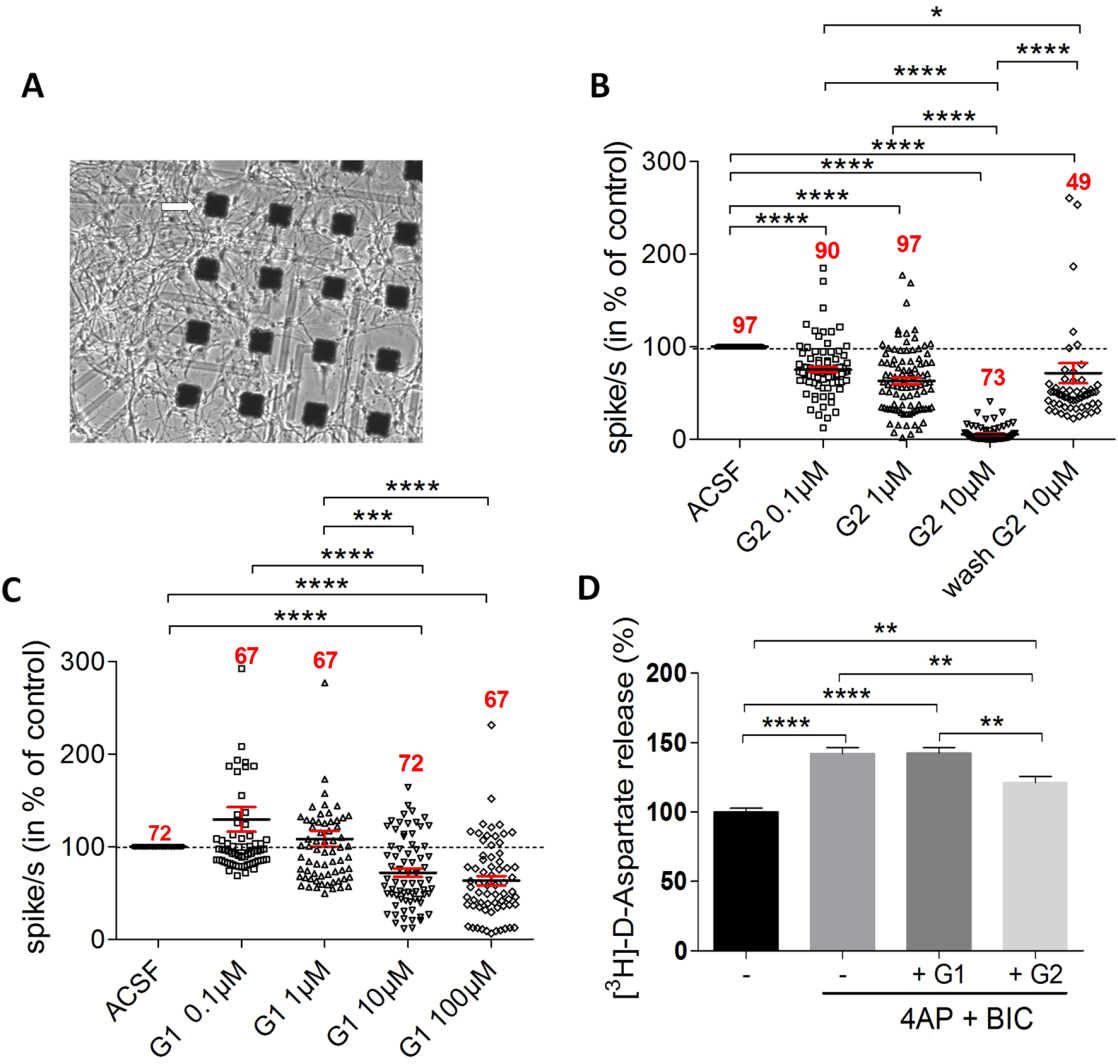
Interfering with 3S-HS with G2 peptide inhibits neural activity and synaptic exocytosis. (**A**) Photomicrograph of growing neuronal cells on few representative electrodes (arrow) from the 64-mutielectrode array (MEA) device. (**B**) pooled recordings of electrode activity (in spikes/sec) (compilation of electrode recordings from n = 5 independent experiments) using MEA. G2 peptide silences, in a dose-dependent manner, and reversibly, the firing activity of neuronal network. Dunn’s multiple comparison test: ****p < 0.0001, *p = 0.0401. (**C**) by comparison to G2 in (**B**), G1 peptide displays a significant though moderate, and non-dose dependent, inhibition of network activity (compilation of electrode recordings from n = 3 independent experiments). Dunn’s multiple comparison test: ****p < 0.001, ***p = 0.0006. Numbers in red indicate the total number of electrode recordings in each condition. (**D**) treatment with G2 peptide decreases evoked glutamate release from hippocampal cell synapses. In contrast, G1 has no effect on synaptic glutamate release (n = 5 independent experiments). Tukey’s multiple comparison test: ****p < 0.0001; control basal versus G2, **p = 0.0013; control 4AP + BiC versus G2, **p = 0.0022; G1 versus G2, **p = 0.0012.

To compare the role of G1 and G2 in neuronal activity, we recorded the firing activity of the hippocampal neuronal culture upon exposure with one or the other peptides using a 64-channel multielectrode array (MEA) system (Fig. 5A). As expected, our dissociated hippocampal culture showed neuronal network synchronized activity at DIV20 revealing network functional connectivity. G2 decreased significantly and in a dose-dependent manner the firing rate by 30% at 0.1 µM, 40% at 1 µM and almost by 100% at 10 µM (Fig. 5B). Interestingly, the effect of G2 at 10 µM was significantly reversed after perfusing cell cultures with neutral control medium. By comparison, G1 significantly reduced the firing frequency by 30% at 10 μM and did not further decrease the firing frequency at 100 μM, indicating a moderate and saturating effect of this peptide (Fig. 5C). These data suggest that G1 and G2 have not a common mechanism for neuronal activity modulation or, if it is the same, that G2 is more effective than G1. To examine the peptides effects on exocytosis at synapses, we used mature hippocampal neurons that were acutely exposed to 4-aminopyridine (4-AP)/Bicucullin to promote evoked release of glutamate^43^ (Fig. 5D). Under these conditions allowing analysis of exocytosis at synapse, G2, but not G1, significantly reduced glutamate exocytosis (by 21%). These results suggest that, in mature hippocampal cells in vitro*,* sulfated HS subtypes recognized by G2 and those recognized by G1 are differently implicated in the maintenance of the neuronal network activity and in the modulation of glutamate exocytosis at synapse.

### Chronic inhibition of 3S-HSs alters synaptic formation

**Figure 6:**
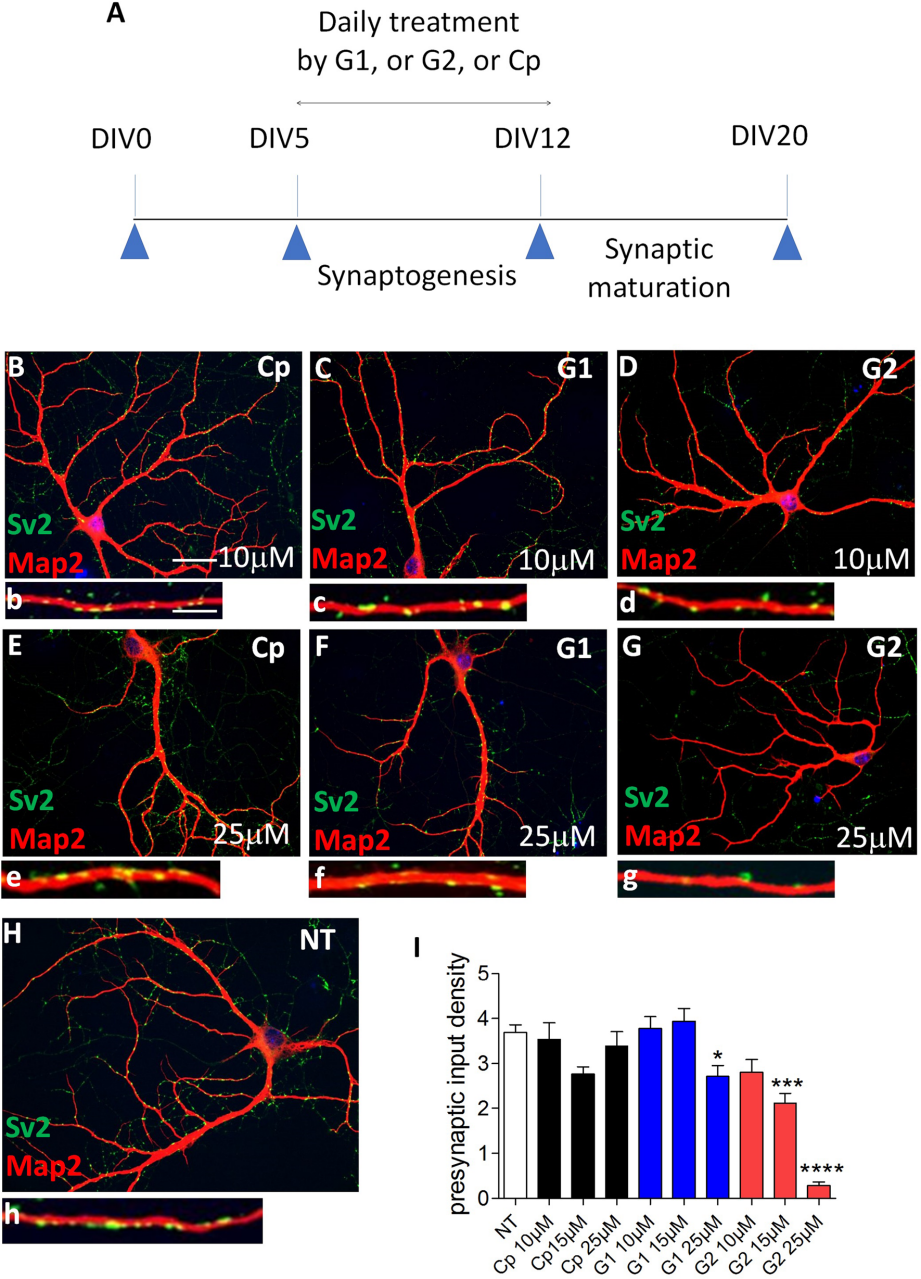
Inhibiting 3S-HS by G2 peptide impairs synapse formation. (**A**) graphical sketch of the daily treatment (DIV 5 to DIV 12) of immature primary hippocampal neurons in culture with G1, G2, or control Cp peptides. (**B**–**G**) co-immunofluorescent neurons labelled with Map2 (red) and Sv2 (green) after treatment with peptides at 10 µM or 25 µM, and H, in non-treated cell cultures (NT). A high magnification of a dendrite bearing Sv2-immunoreactive puncta is shown for each condition (**b**–**h**). (**I**) quantification of the density of presynaptic Sv2-immunoreactive puncta (n = 2 independent experiments). Dunn’s selected comparison test: G2, 25 µM versus NT, ****p < 0.0001; G2, 15 µM versus NT, ***p = 0.0006; G1, 50 µM versus NT, *p = 0.0114. Bar in (**B**), 75 µm (applies for **B**–**H**); Bar in (**b**), 5 µm, (applies for **b**–**h**).

Our proteomic analysis also displayed “synaptic assembly” as a process preferentially associated with proteins recovered by G2 pull-down. To explore it further, we chronically treated hippocampal cell cultures with either G1, G2 or Cp (a control peptide that does not bind to HSs^26^). Hippocampal cells were daily treated with the peptides for 7 days between DIV5 and DIV12, a period when most synapses are formed in vitro (Fig. 6A–I). Treatments by G2 strongly reduced presynaptic boutons density at the surface of hippocampal cell dendrites and cell bodies by comparison to other conditions, as revealed by co-IF with Sv2 and Map2 (Fig. 6G). IF signal quantifications (Fig. 6I) indicated that Cp treatment has no effect while G1 only slightly decreased synaptic density at 25 µM. G2 significantly reduced presynaptic contact density at 15 µM and its maximal effect was reached by 25 µM without sign of neuronal alteration at the concentration used. Consistently, neuronal treatments with peptides at various concentrations did not produce recordable dying cells ruling out an indirect toxic effect of the peptides on synapse formation (Supplementary Fig. S4, Supplementary file). To resume, both G1 and G2 peptides allowed for the identification of several HSPG at the synaptic surface but may differ in their sensitivity to recover these HSPG and some associated signaling molecules. In addition, sulfated HSs recognized by G2 appeared more efficient than those recognized by G1 in regulating functions related to the neural activity and synaptic assembly.

## Discussion

The purpose of our study was to characterize some signaling components and functions associated to 3S-HSs in relation to neuronal development. We achieved this by using peptides which inhibit HSV-1 cell entry by interacting with non-3-*O*-sulfated HSs (peptide G1) or gD-type 3S-HSs (peptide G2)^26^. Several of our observations support the previously established specificity of these peptides^18,26,28,30^ and thus the relevance of using them to target HSs. First, the recovery by pull-down assay of several HSPG and HBPs from the synaptosome surface are consistent with the capacity of G peptides to interact with cell surface HSs^26,30^. Next, it is relevant that nearly 20% of the proteins recovered by G peptides in our experiments are known to be in the HSV-1 signaling pathway including host proteins involved in virus replication. This confirmed the tight link between HSs serving as receptors for HSV-1 (i.e. gD-type 3S-HSs) and the HSV-1 inhibitory peptides capable to bind them^15,26^. Our results by Elisa assay also corroborates precedent indications that G1 and G2 recognize different sites on HSs chains^26^ and meet the expectation that G2 compete with the Hs4c3 Ab which preferentially recognizes 3S-HSs^31^. Finally, the G2 peptide concentration thresholds which trigger in our experiments the inhibition of neuronal activity (0.1 µM) and synaptic assembly (15 µM) are in the range of peptide concentrations generally considered as producing specific effects. In this regard and for comparison, the G peptide concentration range we used was lower than this previously used to block the cellular entry of HSV-1 (10–100 μM)^26^. Finding here that Hs3st2 and Hs3st4 as well as their product 3S-HSs were expressed in developing synapses, in addition to be expressed at other neuronal locations, indicate that synapses have the potential for HS 3-*O*-sulfation during their differentiation and maturation. The detection of Hs3st2 and Hs3st4 in hippocampal neurites suggest that these HS 3-*O*-sulfotransfereases can be transported to synapses by Golgi vesicles which are known to mediate synaptic proteins delivery both in axons and dendrites during neuronal development and synaptogenesis^44^. This may be functionally relevant considering the remarkably stronger expression of Hs3st2 and Hs3st4 during synaptogenesis than at any other time in the course of life in mice^19^. Pull-down experiments using G2 peptide to target specifically the gD-type 3S-HSs produced by Hs3st2 and Hs3st4, and the G1 peptide targeting HSs lacking 3-*O*-sulfates^26^, allowed to identify ensembles of proteins more reliably recovered by G2, or by G1. Differences in protein recovery by either peptides, possibly related to protein affinity to HS subtypes, seems validate the hypothesis that 3-*O*-sulfation act endogenously as transducing molecules^45^. Our results provide substantial insights about the nature of the signaling components that may preferentially associate to 3S-HS or HS lacking 3-*O*-sulfates. Pull-down experiments with G peptides allowed high scoring recognition of synaptic HSPG (i.e. Nrxn-1a, Nrxn-1b, Gpc1), with the notable absence of Sdc2 otherwise detected with HS4C3 Ab, and of synaptic HBPs (i.e. Nlgns-2, 3, 4; Ptprd, Thy-1). These HSPG forming complexes with HBPs like Sdc2 with FGFR and Nrxns with Nlgns gave evidences of the interaction of distinct HS subtypes with molecules having prominent signaling roles during synaptic establishment and maturation^6,10,11^. Indeed, Sdc2 drives synapse formation and maturation by coordinating, in a sulfated HS-dependent manner, exocytosis and presynaptic FGFR signaling^6^. In addition, molecular complexes including Nrxns and Nlgns, recruit proteins to promote synapse formation and maturation and to shape the activity of neural circuits^46^. We do not exclude either that other HSPGs not identified in our assays but participating in synaptogenesis^1,4^ may also carry distinct HS subtypes. Identifying signaling proteins was also one of our goals through the pull-down assay designed to indirectly retrieve, from the cell surface, cytoplasmic proteins bound to HSPGs, while limiting the direct association of peptides with intracellular proteins (see Supplementary file). And in fact, the identification of Sdc2 and/or Nrxns-associated signaling molecules (Cask, Elks, Band 4.1)^39,40^ in G1 and G2 outputs suggests that intracellular proteins recovered in our assay may faithfully reflect signaling pathways involving HSPG at the synapse. In addition, the signalosome identified appeared informative and discriminant since the biological processes more specifically associated to proteins recovered by G2 (i.e. synaptic vesicle endo-exocytosis and synaptic assembly) were validated by our functional assays. Of importance indeed was the confirmation that G2 has a higher inhibitory effect than G1 on neuronal excitability, exocytosis of glutamate and synaptogenesis. In addition, the consistency of the effects produced by G2 peptide, although obtained from distinct functional tests, suggests that 3-*O*-sulfation could sustain parameters that, like activity and adhesion, enhance the overall stability of the developing neural network. Other observations may support this hypothesis. First, defective Hs3sts correlate with abnormal synaptic vesicle clustering and abnormal synapse formation in mating circuits of *C. Elegans*^9^. Then, our analysis of the reactome of Hs3st2 and Hs3st4 based on thousands of RnAseq designate “transmission through synapses” and “signaling across the Neurexin and Neuroligin axis” as two most likely biological processes involving these 3-*O* sulfotransferases in vertebrates. Of interest also are the reports that acute digestion of HSs with heparinase-1 in dissociated hippocampal cells affected synaptic scaling and decreases the mean firing rate of neurons^47^. Similarly, conditional deletion of *Ext1*, a gene coding for an enzyme involved in the polymerization of HSs chains, reduces excitatory synaptic function in pyramidal neurons in amygdala^48^. However, in these two studies the suppression of the entire HSs chains precluded further characterization of the type of HSs involved in the studied processes, and of the mechanisms underlying the observed changes. In drosophila indeed, loss of sulfation has more severe consequences for spontaneous neurotransmission and locomotion than simple loss of HS chains^35^. Here, down regulation of neural activity by treating cells with the G2 peptide could mimic the effect of heparinase on the dissociated cells^47^ and therefore may refine our knowledge of HSs role in maintaining neural activity by revealing the implication of 3S-HSs in this process.

Given their central implication in synapse organization, the role of HSPG and HSs are particularly scrutinized in the context of neuronal and synaptic deficits. Impaired HS supply is associated with autistic-like behaviors in mice^48^, and genetic association has been found between autism and a variant of Hs3st^49^ suggesting that HS could be involved in ASDs. Work from our laboratory has also provided evidences in the last few years for an implication of 3S-HSs and Hs3st2 in Alzheimer Disease (AD)-related tauopathy^21^. In addition, HSV-1 infections in the mouse central nervous system produce an AD-like phenotype, notably through Tau hyperphosphorylation, suggesting that HSV-1 is a risk factor for AD^27^. We identified several proteins including Tau, Picalm-1 and CdK5 in G1/G2 protein clusters that are directly involved in AD, such like Picalm-1 which modulates intracellular Tau accumulation^50^. Our results thus agree with the interest of targeting Hs3st and 3S-HS and their relationship with HSV-1 in the modulation of synaptic deficits as those observed in AD, and possibly also in other disorders. In conclusion, by using inhibitors of HSV-1 through HS and 3S-HS we have highlighted the involvement of gD-type 3S-HS in synapse assembly, neural activity and exocytosis, thus reducing the indeterminacy of 3S-HS function in the nervous system. The protein screening performed as part of this work constitute an important source of data for further in detail exploration of 3S-HSs signaling and characterization of their endogenous ligands.

## Methods

For detailed material and methods see Supplementary file.

All methods and experimental protocols were approved by French Ministry of National Education, Higher Education and Research (MENESR) committee Cometh No.16 (APAFIS#8375-2016123120043752).

### Animals

Mice were treated according to the European Directive number 86/609 (EEC Council for Animal protection in Experimental Research and Other Scientific Utilization) and guidelines for the use of laboratory animals (US National Institutes of Health).

### Statistics

GraphPad Prism7 software (San Diego, CA) was used for statistics. Data are depicted as mean ± SEM. Statistical significance of the differences was calculated by using *p* < 0.05 as the minimum level of significance and one-way ANOVA (or a Kruskal Wallis test if data were not normally distributed, as assessed by D’Agostino and Pearson normality test). This was followed, if overall *p* < 0.05, by a multiple group comparison post hoc test as mentioned in figure legends.

## Supplementary information

Supplementary Information.
