# Supplementary material for: 3-O-sulfated heparan sulfate interactors target synaptic adhesion molecules from neonatal mouse brain and inhibit neural activity and synaptogenesis in vitro
Source: Sci Rep. 2020 Nov 5;10:19114. doi: 10.1038/s41598-020-76030-4 (PMC7644699; doi:10.1038/s41598-020-76030-4)
Supplement: Supplementary file 1 — Supplementary Information. [file 41598_2020_76030_MOESM1_ESM.pdf]

Supplementary File

3-O-sulfated heparan sulfates interactors target synaptic adhesion molecules from neonatal mouse brain and inhibits neural activity and synaptogenesis *in vitro*

Auriane Maïza[a], Nazha Sidahmed-Adrar[a], Patrick P. Michel[b], Gilles Carpentier[a], Damien Habert[a], Carine Dalle[b], Walid Redouane[a], Magda Hamza[a], Toin van Kuppevelt[c], Mohand Ouidir Ouidja[a], José Courty[a], Sandrine Chantepie[a], Dulce Papy-Garcia[a], and Olivier Stettler[a,1]

[a]Gly-CRRET-Croissance Cellulaire, Réparation et Régénération Tissulaire, Univ Paris Est Creteil, Gly-CRRET, F-94010 Creteil, France.
[b]Paris Brain Institute, CNRS UMR 7225, INSERM U 1127, Sorbonne Université, Paris, France.
[c]Department of Biochemistry, Radboud Institute for Molecular Life Sciences, Radboud University Medical Centre, Nijmegen, the Netherlands.

[1]To whom correspondence should be addressed. Email : olivier.stettler@u-pec.fr

Supplementary Figures

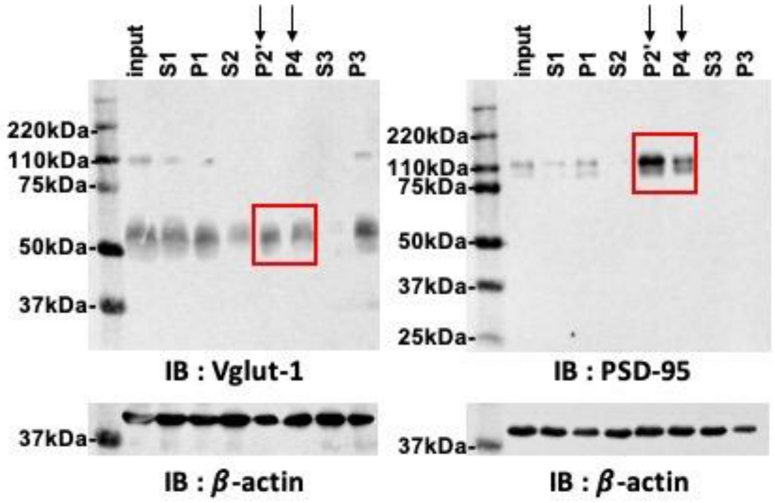

Supplementary Figure S1. Characterization of synaptosomal fractions. Detection of the presynaptic marker Vglut1 and the postsynaptic marker PSD-95 in fractions obtained by differential centrifugation of PND7 forebrain homogenate. S1, 1000 X g supernatant; P1, nuclear pellet; S2, 10,000 X g supernatant; P2', crude synaptosomal pellet; P4, purified synaptosomal pellet; S3, 100,000 X g cytosolic fraction; P3, 100,000 X g particulate fraction. Arrow indicates the fraction used for biochemical experiments and enriched in synaptic markers (red frames).

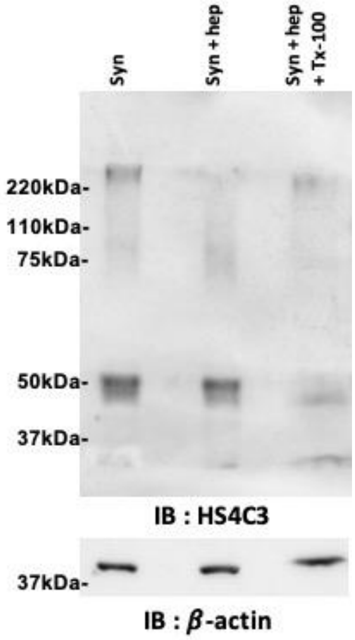

Supplementary Figure S2. Original immunoblot of Figure 1A.

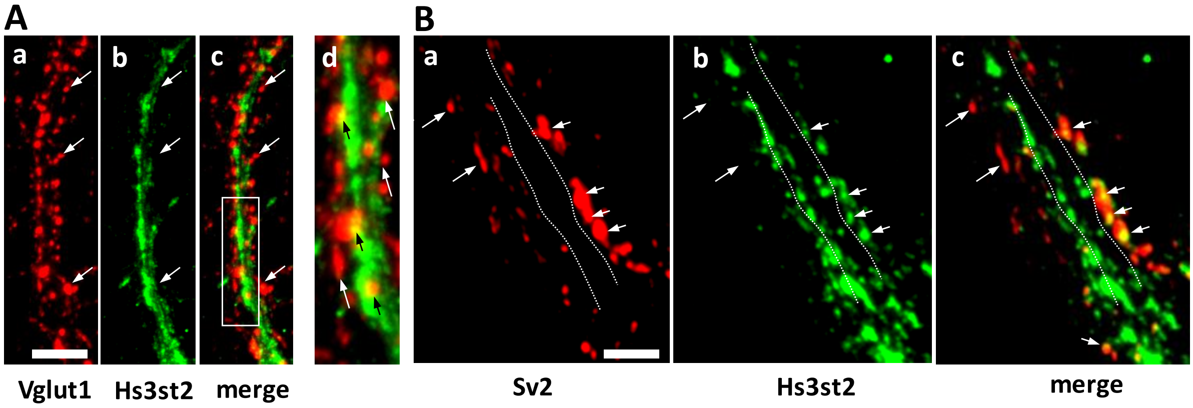

Supplementary Figure S3. Synaptic localization of Hs3st2 in primary hippocampal neurons. A, Hs3st2 distribution (in green) does not match this of the excitatory presynaptic marker Vglut1 (in red) at low magnification (arrows, Aa-c). Higher magnification of the inset in Ac (Ad) reveals partial overlays of Hs3st2 (in green) and Vglut1 IF (in red) at the periphery of neurites (black arrows), while other Vglut1 puncta seem devoid of Hs3st2 labelling (white arrows). B, high magnification of neurites reveals discrete overlays of Hs3st2 (in green) and the universal presynaptic marker Sv2 IF (in red) (short arrows, Bb, c), while other Sv2 puncta seem devoid of Hs3st2 labelling (long arrows, Bb, c). Dashed lines in Ba-c set the distribution limit of Sv2 immunofluorescent puncta alongside neurite. Bar in Aa, 5 µm (applies for Ab,c). Bar in B, 2 µm (applies for Bb, c).

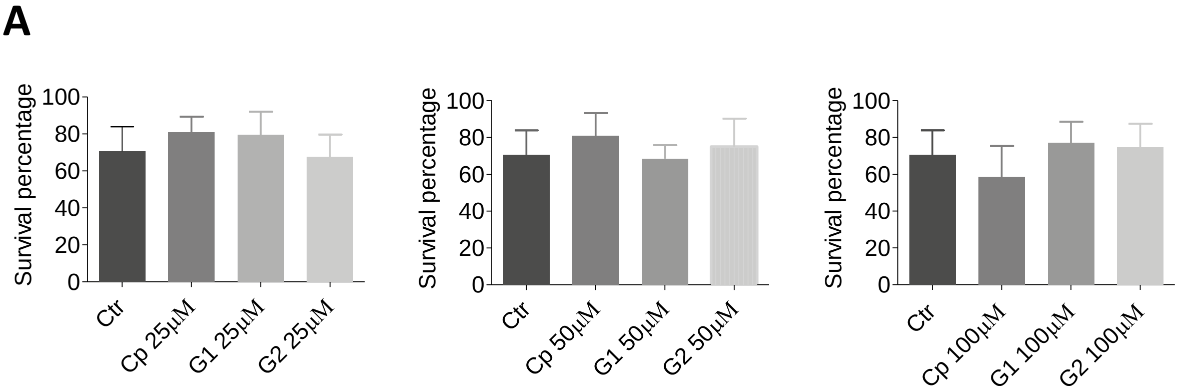

Supplementary Figure S4. Neuronal cell integrity after chronic treatment with G peptides. A, survival test by live-dead assay of hippocampal cells after their daily treatment for seven days with G1 or G2 or Cp peptides at 25 µM, 50 µM, or 100 µM. Ctr, non-treated.

Supplementary Data Files

Raw proteomic data are available via ProteomeXchange with identifier PXD017307

Maïza-et-al-G1G2 synaptosome interactome.
10.6084/m9.figshare.9933206

Maïza-et-al-G1G2 synaptosome interactome-normalized.
10.6084/m9.figshare.9933314

Maïza-et-al-SAB.
10.6084/m9.figshare.9933326

Figure 1 data set
10.6084/m9.figshare.12369188
Figure 5 data set
10.6084/m9.figshare.11808147
Figure 6 data set
10.6084/m9.figshare.11808207

Supplementary Tables

	UniProt access	extended protein name	Short protein name	Functional link with HSV-1
**Acto-myosin**	Q60605<br>Q61879<br>Q8CI43<br>Q99104<br>Q8VDD5<br>Q91Z83<br>Q3THE2<br>Q9QXS6<br>P60766<br>Q3UPH7<br>Q6DFV3	Myosin light polypeptide 6<br>Myosin-10<br>Myosin light chain 6B<br>myosin-Va<br>Myosin-9<br>Myosin-7<br>Myosin regulatory light chain 12B<br>Drebrin<br>Cell division control protein 42 homolog<br>Rho guanine nucleotide exchange factor 40<br>Rho GTPase-activating protein 21	Myl6<br>Myh10<br>Myl6b<br>Myo5a<br>Myh9<br>Myh7<br>Myl12b<br>Dbn1<br>Cdc42<br>Arhgef40<br>Arhgap21	• MyosinVa enhances secretion of HSV-1 and cell surface expression of viral glycoproteins [1]<br>• Non-muscle Myosin-9 is a functional entry receptor for HSV-1 [2]<br>• Actin-binding Protein Drebrin Regulates HIV-1-triggered Actin Polymerization and Viral Infection [3]<br>• Early HSV-1 infection is dependent on regulated Rac1/Cdc42 signaling [4]<br>• Actin' up: HSV Interactions with Rho GTPase Signaling [5]
**Kinases**	Q60737<br>O54833<br>P67871<br>P49615	Casein kinase II subunit alpha<br>Casein kinase II subunit alpha'<br>Casein kinase II subunit beta<br>Cyclin-dependent-like kinase 5	Csnk2a1<br>Csnk2a2<br>Csnk2b<br>Cdk5	• HSV-1 infection stimulates CK2 activity [6]<br>• Herpes simplex virus 1 alters CDK-5 localization, and activity during acute infection in neurons [7]
**Microtubule**	Q8R1Q8<br>O88487<br>P63168<br>Q9D0M5<br>Q8CBY8<br>O35071<br>P28740<br>Q9QXL1<br>Q6PFD6<br>B1AVY7<br>Q61771<br>P10637<br>Q80TV8	Cytoplasmic dynein 1 light intermediate chain 1<br>Cytoplasmic dynein 1 intermediate chain 2<br>Dynein light chain 1 cytoplasmic<br>Dynein light chain 2 cytoplasmic<br>Dynactin subunit 4<br>Kinesin-like protein KIF1C<br>Kinesin-like protein KIF2A<br>Kinesin-like protein KIF21B<br>Kinesin-like protein KIF18B<br>Kinesin-like protein KIF16B<br>Kinesin-like protein KIF3B<br>Microtubule-associated protein tau<br>CLIP-associating protein 1	Dync1li1<br>Dync1i2<br>Dynll1<br>Dynll2<br>Dctn4<br>Kif1c<br>Kif2a<br>Kif21b<br>Kif18b<br>Kif16b<br>Kif3b<br>Mapt<br>Clasp1	• The HSV-1 U(L)34 protein interacts with a cytoplasmic dynein intermediate chain [8]<br>• Function of Dynein and Dynactin in HSV capsid transport [9]<br>• HSV Interactions with the host cytoskeleton [10]<br>• Alzheimer's disease-specific tau phosphorylation is induced by HSV-1 [11]<br>• CLASPs required for HSV-induced stable microtubule formation and virus spread [12]
**Intra vesicular trafficking**	P50396<br>Q61598<br>Q9WV55<br>P60879<br>Q7M6Y3	Rab GDP dissociation inhibitor alpha<br>Rab GDP dissociation inhibitor beta<br>Vesicle-associated membrane protein-associated protein A<br>Synaptosomal-associated protein 25<br>Phosphatidylinositol-binding clathrin assembly protein	Gdi1<br>Gdi2<br>Vapa<br>Snap25<br>Picalm	• Role of Rab GTPases in HSV-1 infection<br>• Viral interactions with host cell Rab GTPases [13, 14]<br>• VAPA interacts with VP16 HSV-1 protein [15]<br>• HSV-1 tegument and envelope proteins and viral particles associate with SNAP-25 [16]<br>• PICALM is involved in the herpes simplex life cycle [17]
**Wnt signaling**	Q02248<br>Q61301<br>P26231	Catenin beta-1<br>Catenin alpha-2<br>Catenin alpha-1	Ctnnb1<br>Ctnna2<br>Ctnna1	• Regulation of Wnt/ß-catenin signaling by HSV [18]
**HSV replication**	Q149L6<br>Q9QYJ0<br>Q01853<br>O54962<br>P70168	DnaJ homolog subfamily B member 14<br>DnaJ homolog subfamily A member 2<br>Transitional Endoplasmic reticulum ATPase<br>Barrier-to-autointegration factor<br>Importin subunit beta-1	Dnajb14<br>Dnaja2<br>Vcp<br>Banf1<br>Kpnb1	• DnaJ protein are involved in HSV DNA replication [19]<br>• Tera/Vcp is essential for the replication of HSV [20]<br>• Banf1 enhances HSV-1 replication [21]

				• Importin is required for nuclear import of HSV proteins and capsid assembly [22]
**Others**	P35564	Calnexin	Canx	• Calnexin associates with the precursors of glycoproteins B, C, and D of HSV-1 [23]
	P70677	Caspase-3	Casp3	• Caspase 3 activation during HSV-1 infection [24]
	P22682	ubiquitin-protein ligase CBL E3	Cbl	• Mediates the Removal of Nectin-1 from the Surface of HSV-1-Infected Cells [25]

Supplementary table S1. “SAB” proteins involved in HSV-1 lifecycle

Cellular components enrichment (fold) (p-value)	G1		G2		Input
	SAB				
	Triton 0.5%	Triton 1%	Triton 0.5%	Triton 1%	
Glutamatergic synapse	7.6 p<0.001	5.7 p<0.001	6.4 p<0.001	5.6 p<0.001	3.5 p<0.001
Presynaptic membrane	13.4 p<0.001	13.9 p<0.001	14.1 p<0.001	16.2 p<0.001	5.1 p<0.001
Postsynaptic density	6.3 p<0.001	3.32 p<0.001	6.2 p<0.001	5.5 p<0.001	3 p<0.001
Neuron projection	4.7 p<0.001	5 p<0.001	4.8 p<0.001	4.1 p<0.001	3.7 p<0.001
Synaptic vesicle	8.6 p<0.001	8.1 p<0.001	7.5 p=0.015	8 p=0.092	4.7 p<0.001
Axon	4.7 p<0.001	5.1 p<0.001	3.6 p=0.003	4.1 p<0.001	3.1 p<0.001
Cytosol	2.1 p<0.001	1.9 P=0.004	1.9 p=0.04	2.1 p<0.001	3.3 p<0.001
Dendritic spine	7.1 p<0.001	-	6.2 p<0.001	-	1.94 p=0.012
GABA-ergic synapse	10.4 p=0.002	-	9.1 p=0.005	9.7 P=0.003	3.8 p<0.011
Postsynaptic membrane	6.1 p=0.011	-	6.3 p=0.005	-	-
Dendrite	3.7 p=0.012	-	3.6 p=0.004	-	1.9 p=0.08
Presynaptic active zone cytoplasmic components	25 p=0.033	-	21.8 p=0.058	-	-

Supplementary table S2A. Cellular component related to the "SAB" fractions

Biological process enrichment (fold) (p-value)	G1		G2		Input
	SAB				
	Triton 0.5%	Triton 1%	Triton 0.5%	Triton 1%	
Synapse organization	18.6 p=0.011	-	-	17.2 P=0.017	-
Modulation of chemical synaptic transmission	11.3 p=0.038	-	-	-	-
Microtubule based movement	-	16.6 p<0.001	-	-	-
Anterograde neuronal dense core vesicle transport	-	88.6 p=0.043	-	-	-
Positive regulation of synaptic vesicle clustering	-	-	74.9 p<0.001	81.3 p<0.001	-
Synaptic vesicle endocytosis	-	-	19.7 p<0.001	16 P=0.026	4.94 p<0.001
Cell-cell adhesion	-	-	10.1 p=0.004	-	-
Synapse assembly	-	-	15,3 p=0.02	16.7 p=0.003	-
Presynaptic membrane assembly	52,8 p=0.01	-	45 p=0.02	48.8 p=0.014	-
Postsynaptic membrane assembly	-	-	40 p=0.033	43.4 p=0.024	2.9 p<0.001
Positive regulation of axonogenesis	-	-	-	25.8 p<0.001	-

Supplementary table S2B. Biological processes related to "SAB" fractions

**HS3ST2**	*GeneNetwork v2.0*
**Reactome**	**P-value**
Transmission across Chemical Synapses	3.4×10^{-5}
Neuronal System	9.7×10^{-5}
Amine ligand-binding receptors	3.7×10^{-4}
Neurotransmitter receptors and postsynaptic signal transmission	6.1×10^{-4}
Serotonin receptors	8.4×10^{-4}
Potassium Channels	8.8×10^{-4}
Neurotransmitter release cycle	1.9×10^{-3}
Inwardly rectifying K+ channels	3.3×10^{-3}
Phase 0 - rapid depolarisation	4.0×10^{-3}
Trafficking and processing of endosomal TLR	4.6×10^{-3}
Class A/1 (Rhodopsin-like receptors)	4.8×10^{-3}
Trafficking of AMPA receptors	5.2×10^{-3}
Glutamate binding, activation of AMPA receptors and synaptic plasticity	5.2×10^{-3}
GPCR ligand binding	7.6×10^{-3}
Na+/Cl- dependent neurotransmitter transporters	8.8×10^{-3}
G protein gated Potassium channels	9.7×10^{-3}
Activation of G protein gated Potassium channels	9.7×10^{-3}

Supplementary table S3A. Pathways associated to HS3ST2 in humans

**HS3ST4**	GeneNetwork v2.0
**Reactome**	**P-value**
Neuronal System	3.4×10^{-5}
Protein-protein interactions at synapses	9.7×10^{-5}
Neurexins and neuroligins	3.7×10^{-4}
Phase 2 - plateau phase	6.1×10^{-4}
Transmission across Chemical Synapses	8.4×10^{-4}
Neurotransmitter receptors and postsynaptic signal transmission	8.8×10^{-4}
Voltage gated Potassium channels	1.9×10^{-3}
Phase 0 - rapid depolarisation	3.3×10^{-3}
Potassium Channels	4.0×10^{-3}
GPCR ligand binding	4.6×10^{-3}
Trafficking of AMPA receptors	4.8×10^{-3}
Glutamate binding, activation of AMPA receptors and synaptic plasticity	5.2×10^{-3}
LGI-ADAM interactions	5.2×10^{-3}
NCAM1 interactions	7.6×10^{-3}
Cardiac conduction	8.8×10^{-3}

Supplementary table S3B. Pathways associated to HS3ST4 in humans

Supplementary References (Table S1)

1. Roberts KL, Baines JD (2010) Myosin Va Enhances Secretion of Herpes Simplex Virus 1 Virions and Cell Surface Expression of Viral Glycoproteins. J Virol 84:9889–9896. https://doi.org/10.1128/JVI.00732-10

2. Arii J, Goto H, Suenaga T, et al (2010) Non-muscle myosin IIA is a functional entry receptor for herpes simplex virus-1. Nature 467:859–862. https://doi.org/10.1038/nature09420

3. Gordón-Alonso M, Rocha-Perugini V, Álvarez S, et al (2013) Actin-binding Protein Drebrin Regulates HIV-1-triggered Actin Polymerization and Viral Infection. J Biol Chem 288:28382–28397. https://doi.org/10.1074/jbc.M113.494906

4. Hoppe S, Schelhaas M, Jaeger V, et al (2006) Early herpes simplex virus type 1 infection is dependent on regulated Rac1/Cdc42 signalling in epithelial MDCKII cells. J Gen Virol 87:3483–3494. https://doi.org/10.1099/vir.0.82231-0

5. Van den Broeke C, Favoreel HW (2011) Actin' up: Herpesvirus Interactions with Rho GTPase Signaling. Viruses 3:278–292. https://doi.org/10.3390/v3040278

6. Koffa MD, Kean J, Zachos G, et al (2003) CK2 Protein Kinase Is Stimulated and Redistributed by Functional Herpes Simplex Virus ICP27 Protein. J Virol 77:4315–4325. https://doi.org/10.1128/JVI.77.7.4315-4325.2003

7. Mostafa HH, van Loben Sels JM, Davido DJ (2015) Herpes Simplex Virus 1 Upregulates p35, Alters CDK-5 Localization, and Stimulates CDK-5 Kinase Activity during Acute Infection in Neurons. J Virol 89:5171–5175. https://doi.org/10.1128/JVI.00106-15

8. Ye G-J, Vaughan KT, Vallee RB, Roizman B (2000) The Herpes Simplex Virus 1 UL34 Protein Interacts with a Cytoplasmic Dynein Intermediate Chain and Targets Nuclear Membrane. J Virol 74:1355–1363. https://doi.org/10.1128/JVI.74.3.1355-1363.2000

9. Döhner K, Wolfstein A, Prank U, et al (2002) Function of Dynein and Dynactin in Herpes Simplex Virus Capsid Transport. Mol Biol Cell 13:2795–2809. https://doi.org/10.1091/mbc.01-07-0348

10. Lyman MG, Enquist LW (2009) Herpesvirus Interactions with the Host Cytoskeleton. J Virol 83:2058–2066. https://doi.org/10.1128/JVI.01718-08

11. Wozniak MA, Frost AL, Itzhaki RF (2009) Alzheimer's Disease-Specific Tau Phosphorylation is Induced by Herpes Simplex Virus Type 1. J Alzheimers Dis 16:341–350. https://doi.org/10.3233/JAD-2009-0963

12. Naghavi MH, Gundersen GG, Walsh D (2013) Plus-end tracking proteins, CLASPs, and a viral Akt mimic regulate herpesvirus-induced stable microtubule formation and virus spread. Proc Natl Acad Sci 110:18268–18273. https://doi.org/10.1073/pnas.1310760110

13. Raza S, Alvisi G, Shahin F, et al (2018) Role of Rab GTPases in HSV-1 infection: Molecular understanding of viral maturation and egress. Microb Pathog 118:146–153. https://doi.org/10.1016/j.micpath.2018.03.028

14. Spearman P (2018) Viral interactions with host cell Rab GTPases. Small GTPases 9:192–201. https://doi.org/10.1080/21541248.2017.1346552

15. Oh HS, Knipe DM (2015) Proteomic analysis of the herpes simplex virus 1 virion protein 16 transactivator protein in infected cells. PROTEOMICS 15:1957–1967. https://doi.org/10.1002/pmic.201500020

16. Miranda-Saksena M, Boadle RA, Aggarwal A, et al (2009) Herpes Simplex Virus Utilizes the Large Secretory Vesicle Pathway for Anterograde Transport of Tegument and Envelope Proteins and for Viral Exocytosis from Growth Cones of Human Fetal Axons. J Virol 83:3187–3199. https://doi.org/10.1128/JVI.01579-08

17. Carter CJ (2010) APP, APOE, complement receptor 1, clusterin and PICALM and their involvement in the herpes simplex life cycle. Neurosci Lett 483:96–100. https://doi.org/10.1016/j.neulet.2010.07.066

18. Zwezdaryk KJ, Combs JA, Morris CA, Sullivan DE (2016) Regulation of Wnt/β-catenin signaling by herpesviruses. World J Virol 5:144. https://doi.org/10.5501/wjv.v5.i4.144

19. Eom C-Y, Lehman IR (2002) The human DnaJ protein, hTid-1, enhances binding of a multimer of the herpes simplex virus type 1 UL9 protein to oris, an origin of viral DNA replication. Proc Natl Acad Sci 99:1894–1898. https://doi.org/10.1073/pnas.042689499

20. Huryn DM, Kornfilt DJP, Wipf P (2019) p97: An Emerging Target for Cancer, Neurodegenerative Diseases, and Viral Infections. J Med Chem. https://doi.org/10.1021/acs.jmedchem.9b01318

21. Oh HS, Traktman P, Knipe DM (2015) Barrier-to-Autointegration Factor 1 (BAF/BANF1) Promotes Association of the SETD1A Histone Methyltransferase with Herpes Simplex Virus Immediate-Early Gene Promoters. mBio 6:. https://doi.org/10.1128/mBio.00345-15

22. Döhner K, Ramos-Nascimento A, Bialy D, et al (2018) Importin α1 is required for nuclear import of herpes simplex virus proteins and capsid assembly in fibroblasts and neurons. PLOS Pathog 14:e1006823. https://doi.org/10.1371/journal.ppat.1006823

23. Yamashita Y, Yamada M, Daikoku T, et al (1996) Calnexin Associates with the Precursors of Glycoproteins B, C, and D of Herpes Simplex Virus Type 1. Virology 225:216–222. https://doi.org/10.1006/viro.1996.0590

24. Kraft RM, Nguyen ML, Yang X-H, et al (2006) Caspase 3 activation during herpes simplex virus 1 infection. Virus Res 120:163–175. https://doi.org/10.1016/j.virusres.2006.03.003

25. Deschamps T, Dogrammatzis C, Mullick R, Kalamvoki M (2017) Cbl E3 Ligase Mediates the Removal of Nectin-1 from the Surface of Herpes Simplex Virus 1-Infected Cells. J Virol 91: https://doi.org/10.1128/JVI.00393-17

Supplementary Materials and Methods

Peptides used for assays and treatments

G1 and G2 peptides are 12-mer peptides previously characterized by phage display [26] and shown to block herpes simplex virus entry [26, 28]. Peptide G1 (LRSRTKIIRIRH) recognizes HSs lacking 3-*O*-sulfates, and peptide G2 (MPRRRRIRRRQK) recognizes 3-*O*-sulfated HSs. A 12-mer peptide (named Cp peptide) that does not recognize HSs (RVCGSIGKEVLG) was used in addition as a control [26]. Peptides were laboratory-made on a matrix solid phase, purified by HPLC, and identified by mass spectrometry (Synapt G2 S – Waters). To label peptides either

with a fluorochrome (AlexaFluor-546 C5 maleimide; Fisher Scientific, 10503243), or biotin (biotin-maleimide Sigma, B1267), a cysteine was added at their NH_2 extremity. This allowed the complexing reaction between the cysteine sulfhydryl and maleimide group of AlexaFluor or Biotin.

Primary culture of murine Hippocampal cells

Primary cultures of hippocampal cells were prepared as previously described [51]. Primary hippocampal cell cultures were prepared from E16.5 embryonic C57Black6J mice (Janvier Labs). Briefly, hippocampi were dissected out in Neurobasal medium (Gibco, 21103-049) and the meninge removed. Hippocampi from embryos were collected, washed twice with HBSS (Gibco, 14170-088), and incubated for 10–12 minutes in 2.5% trypsin/EDTA with 2.5% DNAse in HBSS at 37°C. The cells were then dissociated by trituration and plated at a density of 2 500 cells/cm^2 to 35 000 cells/cm^2 on poly-D-lysine-coated (Sigma, P7280) glass coverslips. Plating culture medium consisted of Neurobasal medium supplemented with 2% B27 (Life Technologies, 17504044, Gaithersburg, MD), GlutaMAX TM-I (Gibco, 35050-038), and 0.1% penicillin-streptomycin (Life Technologies, 15140-122). Cultures were maintained at 37°C in a humidified 5% CO_2 atmosphere. After 2 to 24 hours *in vitro*, plating medium was replaced by glial cell-conditioned medium.

Primary culture of cortical glial cells

Primary cultures of glial cells were prepared from PND4.4 Sprague-Dawley rats (Janvier Labs). Cortices were dissected out and processed as previously described [51]. Cells were plated in 75 cm^2 flasks in Dulbecco's Modified Eagle Medium with 4.5 g/L glucose and glutamine (DMEM High glucose GlutaMAX TM-I, Gibco, 61965-026) supplemented with 10% fetal bovine serum

(FBS, Gibco, 10270-098) and 0.1% penicilin-streptomycin (Life technologies, 15140-122). Cells were let to grow until they were sub-confluent (80%) and passaged to 1:2 in same medium. Passages #1 and #2 were used to prepare conditioned medium as followed: DMEM medium was replaced by pre-warmed Neurobasal medium supplemented with 2% B27, GlutaMAX TM-I and 0.1% penicillin-streptomycin for 24h, then filtered and used to feed primary hippocampal neurons. The conditioned medium (CM) was not replaced over the time of the hippocampal culture.

Mice brain synaptosomes preparation

Synaptosomes were prepared according to the method of Pérez-Otaño (https://www.yumpu.com/s/Gqe6qgpbLDYz8scU) modified from this of Ehlers [52]. Four to six forebrains from PND7 or alternatively PND9 mice were homogenized by 20 strokes in a buffer containing sucrose 0.32 M (VWR, 27480.294), HEPES 4 mM (pH 7.4, Gibco, 15630-056), EGTA (Sigma, E4378), EDTA (MPBio, 194 822), protease inhibitors, phosphatase inhibitor cocktail and PMSF (Sigma, 93482). Alternatively, for pull-down assays, HEPES buffer was free of protease and phosphatase inhibitors. Homogenates were centrifuged at 1000 x g for 5 minutes, the "nuclear pellet" (P1) was discarded and the supernatant S1 was recentrifuged at 10,000 x g. Then, the supernatant (S2) was centrifuged at 35,000 rpm for one hour to give the cytosolic fraction (S3) and the microsomal fraction (P3). The P2 fraction called "crude synaptosomal pellet" was resuspended in homogenization buffer and recentrifuged to the give the "washed crude synaptosomal fraction" (P'2). The P'2 pellet was resuspended in homogenization buffer and gently layered on a cushion of sucrose 1.2 M before to be centrifuged at 35,000 rpm for 20 minutes. The interphase was recovered, mixed with homogenization buffer and gently layered on a sucrose cushion of 0.8 M before to be

centrifuged at 35,000 rpm for 20 minutes. The pellet containing “pure synaptosomes” (i.e. the P4 fraction) was recovered and resuspended in homogenization buffer or alternatively in Artificial CerebroSpinal Fluid buffer ACSF (NaCl 140 mM; KCl 2.5 mM; $MgCl_2$ 1 mM; $CaCl_2$ 2 mM; Glucose 10 mM and HEPES 10 mM), or in PBS depending on its subsequent use.

Synaptosomes biotinylation

Pure synaptosomes were washed with PBS and then biotinylated with sulfo-NHS-SS-biotin according to the manufacturer (Thermo Scientific, 89881). Briefly, quenching solution was added to synaptosomes samples that were subsequently centrifuged. Synaptosomes were then lysed using lysis buffer (from Thermo scientific kit) containing protease inhibitors, under sonication and by vortexing. The obtained lysate was centrifuged, and the supernatant layered in a column containing NeutrAvidin agarose. After several rinses, the mix was incubated at room temperature. Then, after another sequence of centrifugation and washing, bound proteins were eluted using a solution containing SDS-PAGE buffer and were prepared for western blot analysis.

Hippocampal cell treatments

To digest out HSs chains, hippocampal cells were treated for 6 hours at 37°C with a cocktail of heparitinases (Sigma, H2519, H6512, H8891) directly mixed-up with the conditioned medium. In other experiments, HPLC grade peptides G1, G2 and control peptide CP, were used for hippocampal cell treatment. Cells were daily treated for 7 days between DIV5 and DIV13 with either 10, 15, 25 µM. At the end of the treatment period, hippocampal cells were fixed for 10 minutes in 4% paraformaldehyde in PBS (Gibco, 14190-094) then subsequently incubated in

50 mM NH_4Cl for additional 10 minutes, and finally rinsed several times in PBS for subsequent immunofluorescent experiments.

Synaptic proteins pull-down

Pull-down assay with biotinylated peptides was adapted from [53] and allowed the recovery of membrane proteins together with their intracellular signaling molecules. Pure P4 synaptosomes fractions were resuspended in ACSF and incubated for 45 min at room temperature with 10 µM biotin-G1 or biotin-G2 or ACSF only as control. The synaptosomes were centrifuged and washed thoroughly by multiple rinses performed with large volumes of PBS in order to remove unbound peptides. Synaptosome pellets were then lysed by incubation for 5 min in lysis buffer preserving the initial interactions of peptides with membranes (containing 20 mM Tris-HCl pH 7.6, 150 mM NaCl, 5 mM $MgCl_2$, 0.2 mM PMSF, 1% protease inhibitors, 1% phosphatase inhibitors and including either 0.5 or 1% Triton-X100). The lysed fractions were then centrifuged at 13,000 rpm for 10 min and the supernatants were recovered. 400 µg (for SynA experiment) or 200 µg (for SynB experiment) of synaptosome lysate proteins were incubated overnight with washed Streptavidin-Sepharose (GE Healthcare, Life Sciences, 17-5113-01) under agitation. Sepharose beads were extensively washed with 3 x 15 mL of cold PBS. Finally, Sepharose bead in pellets were washed with water, centrifuged and re-suspended with Laemmli buffer (Bio Rad, 161-0747).

Immunofluorescence

For immunofluorescence experiments, cells were fixed with either methanol (MeOH) or paraformaldehyde 4% (PFA) depending on the experiment. For methanol fixation, cells were washed with PBS and then let for 10 minutes in MeOH, before being rinsed in PBS. For PFA

fixation, cells were let for 10 minutes at room temperature and then in 50 mM NH_4Cl for 10 minutes to avoid aldehyde groups autofluorescence and rinsed in PBS. For immunofluorescence labelling, cells were washed with PBS or alternatively with PBS containing 0.05% Triton-x100. Unspecific sites were blocked with PBS (or PBS-Triton) containing 2% fetal bovine serum (FBS) and then cells were incubated in the blocking solution overnight at 4°C with one of two of the following antibodies: Hs3st2 (Santacruz, sc-107228), Hs3st4 (Abcam, MAB6085, R&D), HS4C3 (non-commercial, see ref. 31), Neurofilament (DSHB, 2H3), Vglut1 (a gift from Dr. El Mestikawy), MAP2 (Abcam, Ab24640), SV2 (DSHB), PSD95 (Thermo Scientific, 6G6-1C9) and Homer (Thermo Scientific, PA5-21487). Cell were then washed with PBS (or PBST) and incubated for one hour with the secondary anti-rabbit (Life technologies, A31572 or A21206) or anti-mouse (Life technologies, A31570 or 21202). After washing with PBS, nuclei of cells were staining with DAPI and washed with PBS before to be mounted with Prolong Gold antifade reagent (Life technologies, P36934).

Immunoprecipitation

The mouse monoclonal antibodies Hs3st2 (Sigma; WM0009956), Hs3st4 (R&D interchim; MAB60851), and HS4C3 Ab [31] or the rabbit polyclonal antibody Syndecan-2 Ab (Biorbyt, orb13481), were mixed with intact synaptosomes (40 µg), together with protein G (MACS Miltenyi Biotec, 130-071-101). The mixture was mixed and incubated for 30 minutes on ice. Microcolumns (MACS Miltenyi Biotec) were washed with lysis buffer containing phosphatase inhibitors (Sigma, P5726, P0044), PMSF, proteases inhibitor (Sigma, P8340), 150 mM Tris, Deoxycholate (Sigma, D-6750), Triton X 100 (pH 7.4). Column were loaded with the mix containing samples, antibody, and protein G, and washed with RIPA buffer (Thermo Scientific, 89901). Then, columns were washed with low salt buffer containing Igepal (Sigma Aldrich, G

13021), and Tris (pH 8). Low salt buffer was replaced by warmed Laemli buffer containing beta-Mercaptoethanol (Sigma, M6250) allowing elution of bound proteins.

Image acquisition and analysis

Image acquisition was performed using an Olympus IX81 inverted microscope equipped with a FV1000 laser scanning unit with a 40 X objective (oil immersion, NA = 1.30). Two lasers were used, a blue laser diode (473 nm) for the green labeling, and a green HeNe gas laser (543 nm) for the red labeling. Stacks of images were acquired with a sampling rate of 70 nm/pixel (2D spatial resolution, 130 nm, after image deconvolution), and a z step of 0.5 µm. Analysis of SV2 presynaptic boutons were realized from images acquired with a DSU spinning-disk Olympus IX 81 inverted microscope with an immersion 40x objective (oil immersion, NA = 1.25) and processed with image-J. Map2 immunoreactive neurons were silhouetted and clusters of Sv2 IF contacting dendrites and cell bodies were quantified visually and manually. A total of 20 neurons per condition from two independent experiments were analyzed.

Multielectrode arrays

Electrophysiological recordings were realized from primary cultured hippocampal cells that have grown on a 64-channel multielectrode array (MEA) for 20 days, at 37°C in a humidified atmosphere of 5% CO^2. MED64 probe (P515A, Alpha-Med Sciences) containing a grid of 64 planar electrodes was coated with poly-D-lysine and PEI (polyethyleneimine). Hippocampal neurons were plated in MED64 at a density of about 300 000 cells and cultured with the same media than this used for the cultures in other assays. Spike activity was recorded in primary hippocampal culture at DIV20. For MEA recording, the media in the MED probe was replaced with the extracellular solution (composition above) and allowed to re-equilibrate for

approximately 5 min before the beginning of the recording. Data were amplified 1000 times and sampled at 20 kHz. Data were filtered with a low-cut filter of 100 Hz. Mobius software (Alpha Med Scientific) was used to serially detect spikes by threshold crossing of high pass filtered data. The threshold was set individually for each electrode at five S.D. above the average RMS (root mean square) noise level. MEA data were quantified using the number of spikes detected in each electrode of the MEA for each second of recording. The average of spike/s for 10 minutes s of recordings was used as a measure of electrical activity. Other offline data analyses were performed using Microsoft Excel, Origin and SigmaPlot software. Each recording corresponding to a given pharmacological condition was done over a 10 minutes time period with perfusion of extracellular solution at a flow rate of 2 ml/min at 37°C. Data were pooled from 3 to 5 independent experiments for quantification and evaluation of statistical significance.

Glutamate release assay

[2,3-^{3}H]-D-aspartate, a non-metabolizable analog of L-glutamate that labels the cytosolic and vesicular pools of endogenous excitatory amino acids, was used to estimate the release of glutamate as described before [43]. Briefly, the culture medium of hippocampal cultures was rapidly removed, and the cultures maintained for the next 30 min in an ACSF buffer containing 0.5 µl [2,3-^{3}H]-D-aspartate (13 Ci/mmol; Perkin Elmer, Waltham, MA; NET581250UC). After a double wash to eliminate the excess of radiochemical not incorporated by hippocampal neurons, the cultures were submitted to a depolarizing treatment with 4-aminopyridine (4-AP; 2.5 mM; Sigma Aldrich; A7,840-3) and bicuculline (Bic; 50 µM; Sigma Aldrich; 14340) in the presence or not of the G1, or G2 peptides using the same ACSF buffer as before. Control cultures did not receive the depolarizing treatment after D-aspartate loading. The evoked

released of D-aspartate was estimated by measuring the radioactivity accumulating in the incubation medium after 30 minutes of stimulation. Radioactivity measurements were made with a Tri-Carb 4910TR liquid scintillation counter (Parkin-Elmer, Villebon sur Yvette, France). Data were analyzed using Microsoft Excel and Prism 7 software.

Western blot

Proteins in synaptosome samples or in IP eluates were separated by electrophoresis on 10% reducing acrylamide gels. Proteins were then transferred to PVDF membranes using Trans-Blot Turbo Transfer System (Bio-Rad). Membrane were blocked in PBS containing 0.01% Tween-20 (PBST) and 3% milk overnight at 4°C. Primary antibodies used for western immunoblotting were: Hs3st2 (Sigma Aldrich, WH0009956M1), Hs3st4 (R&D, MAB60851), HS4C3 (39), VGlut1 (a gift from Dr El Mestikawy), PSD 95 (Thermo Scientific, 6G6-1C9), and Sdc2 (Biorbyt, Orb13481). Membranes were incubated for 1 hour at RT with antibodies diluted in blocking buffer. Membranes were then washed three times in PBST and subsequently incubated for 1 hour in in PBST containing 1% milk and horseradish peroxidase (HRP)-conjugated secondary anti-mouse (Jackson ImmunoResearch, 715-035-151) or anti-rabbit (Jackson ImmunoResearch, 111-035-144) antibodies. Quantification were done using beta-actin (Sigma, A5316) antibody. Blots were developed with Luminata Forte (Millipore, WBLUF0500) following manufacturer’s instructions.

Binding assays

For ELISA competition assay, 96-well plate (Costar) was coated with Heparin-BSA complex in a buffer containing Tris-HCl, pH 7.4 plus EDTA and incubated overnight at 4°C. After washes with PBS/Tween 20, saturation of heparinized wells was achieved with PBS and BSA (3%) for

1 hour at RT. Plates were then washed several times with PBS/Tween 20. AlexaFluor-G2 (G2-F) alone or G2-F mixed with increasing concentrations of either G2, G1, CP, or HS4C3 Ab was added to well and incubated 1 hour at room temperature and then washed 3 times with PBS/Tween 20. Plates were read by using a fluorimeter (Infinite M100, Tecan). For binding assay on synaptosomes, pure synaptosomes were fixed with 4% PFA and uses in a competition assay after binding of AlexaFluor G2 (1 µM) mixed with increasing concentrations of unlabeled G2 peptide. For all experiments, triplicates were realized for each condition, and blank was subtracted to values. The number of independent experiments varied between 3 and 5 and are indicated in corresponding legends.

Mass spectrometry and proteomic analysis

The migration by electrophoresis on polyacrylamide gel of the protein lysates of the synaptosomes and of the proteins eluted after the pull-down was brief, so as not to completely separate the proteins and avoid dilution in the gel of the least concentrated proteins. Gels were stained with Coomassie Brilliant Blue R-250 (Biorad) and gel bands were excised into small pieces and distained with 50 % acetonitrile (ACN) and 50 mM ammonium bicarbonate (AMBIC, Sigma, 09830). Proteins in gel were reduced with Dithiothreitol, then alkylated with iodoacetamide, and digested with 10 ng/µL Trypsin in 50 mM AMBIC overnight at 37°C. Peptides were eluted twice with 5 % formic acid (FA) and 50 % ACN followed by second elution with ACN. Tryptic peptides were dried by vacuum centrifuge and then resuspended in 0.1% FA for analysis by LC-MS/MS using nanoACQUITY ultra-performance liquid chromatography (nanoUPLC) system (Waters, Milford, USA), coupled to NanoElectroSpray Ionisation hybride Quadrupole Time-of-flight (QTof) tandem mass spectrometer with ion mobility separation (IMS) (SynaptG2-Si, Waters Corporation), managed by MassLynx 4.1

software. Separation of tryptic peptides was performed by reversed phase chromatography using a C18 column (BEH dC18, 1.7 µm, 100 µm x 100 mm, Waters Corporation), by a gradient of 1 to 40 % ACN in 0.1 % FA (mobile phase B) and water with 0.1 % FA (mobile phase A), over 45 min, with column temperature of 40 °C and flow rate of 450 nL/min. The eluted peptides were introduced into the mass spectrometer operated in the positive mode from m/z 50–4000 and operated in DIA mode (mobility Tof resolution) combined with ion mobility separation (MSe) referred to as HDMSE (high-definition MSE). This Technology offers alternating low and high energy scans which was automatically set by ramped collision energies HDMS (20 - 55 V). Proteins are identified using ProteinLynx Global SERVER software version 3.0.3 (PLGS, Waters) and Peak list files were searched against the Mouse UniProt protein database (released on Feb 2018) using the following workflow parameters: setting the peptide and fragment tolerance to automatic, minimum number of fragment ion matches per peptide = 1, minimum number of fragment ion matches per protein = 3, minimum number of unique peptides per protein = 1, one trypsin missed peptide cleavage, static modification of cysteine (carbamidomethylating), variable modifications of methionine (oxidation), and false discovery rate (FDR) maintained at 1%. Obtained data were then analyzed using Funrich software (3.1.3 version) and DAVID (6.8 version). MS was performed from samples obtained from two independent pull-down assays "SynA" and "SynB" with SynB having half the protein concentration than SynA. Samples in SynA and SynB were named "G1", "G2", "untreated". An additional sample in SynB was "input". "G1" and "G2" corresponded to processed samples (see "*Pull-down assay*" in the current section) from synaptosomes exposed either to biotinylated G1 or G2 and lysed either with 0.5 or 1% Triton-X100. "Untreated" (i.e. control) corresponds to processed samples from synaptosomes non-exposed to biotinylated peptides but exposed to streptavidin beads after their lysis (with 0.5% Tx-100). Finally, "input" stands

for pure synaptosomal fraction used in pulldown assay SynB. “G1” and “G2” samples were normalized against “control” in SynA and SynB in order to exclude from subsequent analysis false positive proteins which bind non-specifically to streptavidin in absence of biotin.

Live dead assay

Live dead assay was realized according to manufacturer protocol (Abcam ab115347). Hippocampal cell culture media were aspirated and replaced with 5X dye in HBSS directly to cells. After 10 minutes, lived cells appeared in green and dead cells in red. Live and dead cells were counted manually from 2 independent experiments totalizing 100 cells per condition, and analyzed for statistical significance.
